# Transcriptional evidence of neuroendocrine cell plasticity beyond histological boundaries in lung neuroendocrine neoplasms: an *in-silico* analysis suggesting a progression model

**DOI:** 10.1186/s13046-026-03790-8

**Published:** 2026-07-28

**Authors:** Giuseppe Pelosi, Mauro Papotti, Riccardo Papa, Eleonora Duregon, Maria Gemelli, Sergio Harari, Angelica Sonzogni, Alice Laffi, Tommaso De Pas, Chiara Catania, Barbara Bassani, Fiorenza Lotti, Antonino Bruno, Paola Muti, Fabrizio Bianchi

**Affiliations:** 1 Department of Oncology and Hemato-Oncology, University of Milan, Milan, Italy; 2 Inter-Hospital Pathology Division, Istituto di Ricovero e Cura a Carattere Scientifico (IRCCS) MultiMedica, Milan, Italy; 3 Department of Oncology, University of Turin, Turin, Italy; 4 Unit of Medical Oncology, Istituto di Ricovero e Cura a Carattere Scientifico (IRCCS) MultiMedica, Milan, Italy; 5 Department of Medical Sciences and Community Health, University of Milan, Milan, Italy; 6 Division of Pneumology, Istituto di Ricovero e Cura a Carattere Scientifico (IRCCS) MultiMedica, Milan, Italy; 7 Department of Pathology and Laboratory Medicine, Fondazione Istituto di Ricovero e Cura a Carattere Scientifico (IRCCS), Istituto Nazionale Tumori, Milan, Italy; 8 Division of Medical Oncology, Humanitas Gavazzeni, Bergamo, Italy; 9 Laboratory of Innate Immunity, Unit of Molecular Pathology, Biochemistry, and Immunology, Istituto di Ricovero e Cura a Carattere Scientifico (IRCCS) MultiMedica, Milan, Italy; 10 Department of Biotechnology and Life Sciences, Laboratory of Immunology and General Pathology, University of Insubria, Varese, Italy; 11 Department of Biomedical, Surgical and Dental Health Sciences, University of Milan, Milan, Italy; 12 Istituto di Ricovero e Cura a Carattere Scientifico (IRCCS) MultiMedica, Milan, Italy; 13 Unit of Cancer Biomarkers, Fondazione IRCCS Casa Sollievo della Sofferenza, San Giovanni Rotondo, Italy

**Keywords:** Neuroendocrine, Tumor, Carcinoma, Evolution/plasticty, Immune microenvironment, Epigenetic regulation, Immunosuppressive signaling, Lung

## Abstract

Little is known as to whether there may be a patogenetic continuum among subsets of lung neuroendocrine neoplasms (NENs), including both neuroendocrine tumors (NETs) and neuroendocrine carcinomas (NECs). In this study, transcriptomic data from 205 pulmonary NETs and NECs were collected from four publicly available datasets and analyzed through supervised clustering of a curated 20-gene signature dealing with neuroendocrine differentiation, key oncogenic pathways, chromatin remodeling genes, and lineage-specific transcription factors. Two major clusters were identified, PNEN-A and PNEN-B. Compared to PNEN-A and independently of histological typing, PNEN-B demonstrated reduced neuroendocrine gene expression and increased activation of pulmonary epithelial cell lineages, such as glandular (club/AT2⁺), basal (ΔNp63⁺/p63⁺/CK5⁺), and tuft cell (POU2F3⁺) differentiation. PNEN-B tumors also exhibited features of inflamed but functionally constrained immune microenvironment, with increased expression of immunomodulatory neuropeptides (calcitonin, proopiomelanocortin, and gastrin-releasing peptide), alongside enrichment of T cell exhaustion markers. PNEN-B tumors significantly correlated with poor prognosis in a subset of cases. These findings reveal common transcriptional programs across the entire spectrum of pulmonary NENs, independent of histological typing. Attenuation of neuroendocrine differentiation, activation of alternative cell lineages, and modulation of microenvironment highlight subsets of tumors with potential pathogenetic and clinical implications.

**Supplementary Information:**

The online version contains supplementary material available at 10.1186/s13046-026-03790-8.

## Background

Lung neuroendocrine (NE) neoplasms (NENs) include typical carcinoid (TC), atypical carcinoid (AC), small cell lung carcinoma (SCLC), and large cell neuroendocrine carcinoma (LCNEC). TCs and ACs are low- to intermediate-grade neuroendocrine tumors (NETs), whereas SCLCs and LCNECs are high-grade malignant neuroendocrine carcinomas (NECs) [1]. In these neoplasms, NE differentiation is governed by spatial and temporal dynamics leading to huge inter- and intra-tumor heterogeneity [2]. In this regard, about 10–15% of SCLCs may exhibit a non-NE/NE-low phenotype. Non-NE subpopulations are found within NE-high SCLCs via *MYC/NOTCH* signaling [3], combined SCLCs with squamous cell carcinomas show a YAP1-dependent NE-low transcriptional profile [4], and LCNEC-like SCLC [5] and type II SCLC-like LCNEC exhibit low NE profile along with enhanced NOTCH/immune profile [5]. Furthermore, subsets of non-small cell lung carcinomas (NSCLCs) may converge to NECs via *TP53-RB1* co-inactivation, EGFR-TKI-resistant lung adenocarcinomas may be enriched for the NE determinant VGF [6], some ACs exhibit weak and erratic expression of NE markers, and NSCLC-like LCNECs are characterized by NE-high profiles, *TP53* mutation and *RB1* proficiency [5]. These observations support the concept that NE plasticity may dynamically drive lung carcinogenesis, probably through paracrine, autocrine, and endocrine mechanisms, thereby recapitulating embryogenesis and post-natal life, as NE cells are crucial for tissue homeostasis, injury repair, chronic disease, tumor growth and metastasis formation [7–11]. Of note, a complex crosstalk between NE cells and tumor immune cell microenvironment (TIME) is likely to orchestrate tumor morphogenesis, with NE mediators modulating immune response, immune evasion, and tumor progression [12, 13]. Our working hypothesis discussed in this Commentary is that NE plasticity in lung NENs may be dynamically coupled to, and may actively drive transitions among different tumor types defined by the current WHO classification, through the upregulation of lineage-defining transcription factors (TFs), including *ASCL2, POU2F3, YAP1, TP63, MYC,* and *NOTCH*, resulting in acquisition of non-NE profiles, fate specification, immune-evasive phenotypes, and adverse clinical outcomes.

An *in-silico* study of 205 lung NENs spanning the full disease spectrum, including 93 SCLCs, 40 LCNECs, 47 TCs and 25 ACs from four publicly available data sets [5, 14–16], was conducted to investigate the relationship existing among NE plasticity, associated factors, and immune dysregulation (Supplemental Methods). Analyses were carried out on RNAseq- and microarray-based normalized gene expression data [5, 14] (Supplemental Methods). A manually curated 20-gene signature encompassing key mechanisms of core oncogenic pathways (*MYC*, *NOTCH1*, *TP53*, *RB1*, *SFN*, *ARID1A*, *ARID1B*, *ARID2*, *SMARCA2*, *SMARCA4*, *SMARCB1*), NE differentiation *(CHGA, CHGB, SYN1*, *INSM1) and* lineage-defining TFs (*ASCL1*, ASCL2, *NEUROD1*, *POU2F3*, *YAP1)* was applied as a simplified and practical approach for clustering recurrently altered genes in lung NENs (Supplemental Methods).

**Fig. 1:**
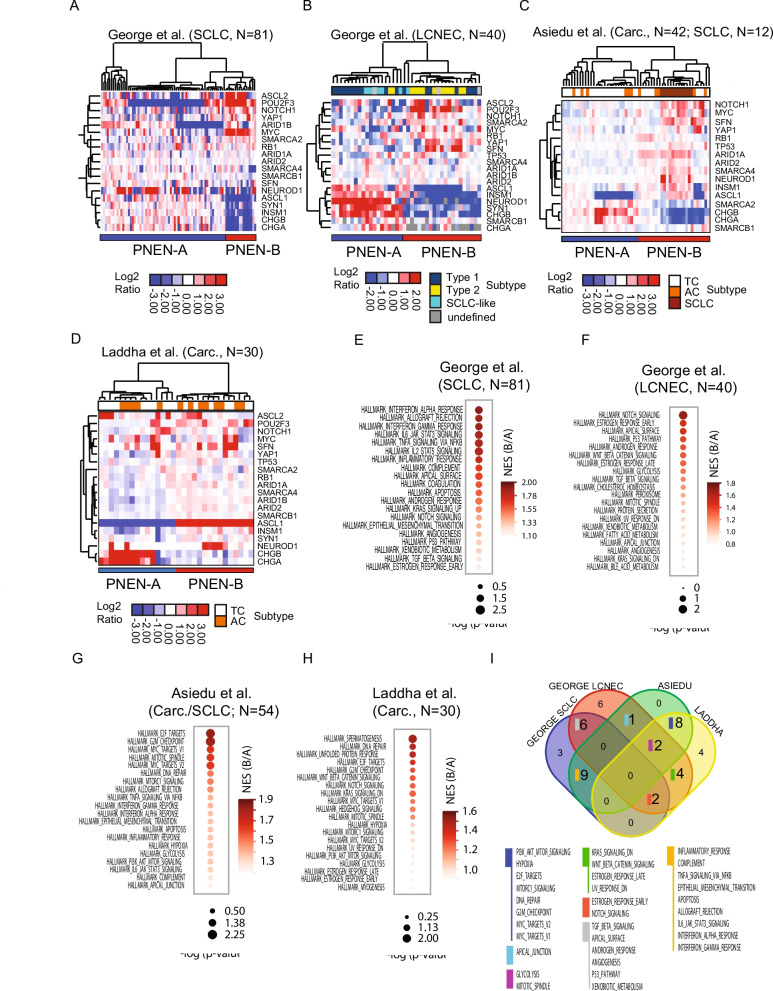
Hierarchical clustering analysis of the 20-gene signature in the George et al. SCLC cohort **A**, George et al. LCNEC cohort **B**, Asiedu et al. cohort **C** and Laddha et al. cohort **D**. Color codes are as per the legend (*i.e.,* median-centered log₂ gene expression ratio). N, number of patients. Tumor classified in PNEN-A and B are also shown underneath the heatmap. **E-H** Bubble plot of the Hallmark GeneSets found enriched in PNEN-B vs. PNEN-A NE tumors in the indicated datasets by Gene Set Enrichment Analysis (GSEA). Colors of the bubbles represent the magnitude of enrichment (NES, normalized enrichment score) and are as per the legend. Size of the bubbles represents the statistical significance of the enrichment (-Log of p-values), *i.e.,* the bigger the most significant and are as per the legend. Y-axes, Gene Sets. **I)** Venn Diagram of enriched Hallmark GeneSets

We identified, in all four datasets, a consistent segregation into two major clusters based on gene expression patterns, namely PNEN-A and PNEN-B, which was independent of the current WHO classification (Fig. 1 A–D). Of note, PNEN-B cluster showed a NE-low profile associated with increased expression of NOTCH1, MYC, TP53, POU2F3, YAP1, RB1, ASCL2, and SWI/SNF complex members (Fig. 1 A–D), and with ASCL1 in carcinoids only (Fig. 1 C, D). Notably, the main structure of the dendrogram in Fig. 1A (SCLC samples) appears to be largely driven by POU2F3 expression, which is strongly upregulated in all PNEN-B samples and, to a lesser extent, in a subset of PNEN-A samples. POU2F3 is a master regulatory transcription factor that defines a distinct, non-neuroendocrine, tuft cell-like variant of SCLC and LCNEC, known as SCLC-P [17] and LCNEC-P [18], respectively. Consistent with these observations, we found that nearly all samples belonging to SCLC-P and LCNEC-P subtypes were classified within the PNEN-B cluster (Supplemental Table 1 and Fig. 1 A, B).

**Fig. 2:**
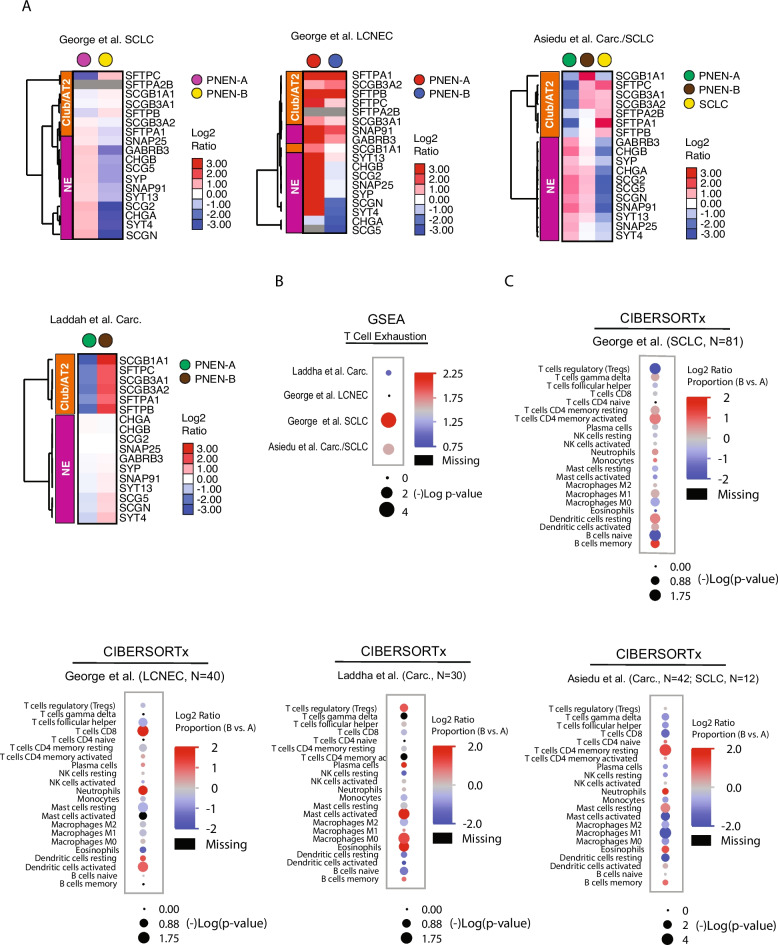
**A** Hierarchical clustering analyses of a cell-of-origin signature (N=18 genes) including NE and Club/AT2 lineage markers, using log 2 ratios of avarage of expression data in pooled PNEN-A and B NE tumors. In Asiedu et al. dataset, we have highlighted the expression profile of genes in SCLC compared to carcinoids in PNEN-A and B. Color codes are as per the legend. **B** Bubble plot of the T Cell Exhaustion signature enriched by GSEA in PNEN-B NE tumors in the indicated datasets (Y-axes). Colors of the bubbles represent the magnitude of enrichment (NES, normalized enrichment score) and are as per the legend. Size of the bubbles represents the statistical significance of the enrichment (-Log of p-values), *i.e.,* the bigger the most significant and are as per the legend. **C** Bubble plot of estimated abundances of TIME cell types by CIBERSORTx analysis, in PNEN-B vs. A NENs. Color codes are as per the legend. Size of the bubbles represents the statistical significance of the enrichment (-Log of p-values), *i.e.,* the bigger the most significant and are as per the legend. Y-axes, Cell Types

Aggressive profile of PNEN-B carcinoids was enriched in immunosuppressive expression of calcitonin (CALCA), proopiomelanocortin (POMC), gastrin-releasing peptide (GRP) and fascin (FSCN1), supporting regulation through hormonal and actin-binding cell motility factors, respectively (Supplemental Figure 1 A). In the NE-low PNEN-B cluster, pathways of inflammatory response, TGFβ/EMT signaling, stemness, and cell proliferation were observed (Fig. 1 E–I), which were further confirmed by clustering custom gene signatures of known cell-of-origin markers, specifically, NE versus club/AT2 cell (Fig. 2A). Furthermore, the NE-low PNEN-B cluster also upregulated basal cell markers DNp63 and CK5 (KRT5), and the tuft cell lineage determinant POU2F3 (Supplemental Figure 1B), consistent with phenotype-switching remodeling [19]. Of note, these NE-low/club AT2-high/YAP1-high tumors bearing RB1 retention, NOTCH activation, MHC/HLA expression (Supplemental Figure 2) and inflamed TIME were similar to about 50% of LCNECs showing SCLC traits with NE^low^ profile [20–22]. In turn, POU2F3 aligned with PNEN-B cluster along with SWI/SNF activation [23] and basal cell marker expression, such as DNp63 and CK5 (KRT5) (Supplemental Figure 1B), consistent with the recent evidence that a CK5-positive basal cell of origin underlies multilinear plasticity in neuroendocrine, tuft, squamous-basaloid and glandular cells [24]. As carcinoids and pure NECs lack DNp63 and basal cell cytokeratins [25, 26], while POU2F3 is detected in a subset of basaloid squamous carcinomas, SCLCs and LCNECs [17, 18], the PNEN-B cluster appears to be a heterogeneous group comprising NETs, NECs and even NSCLCs showing transcriptional traits of adenocarcinoma–, squamous/basaloid cell carcinoma–, and tuft cell like–associated lineages. Mechanistically, Gene Set Enrichment Analysis (GSEA) revealed that lineage-switching phenotyping of PNEN-B neoplasms was enriched in IFNα/IFNγ response (Fig. 1 E, G) and T-cell exhaustion signatures [27] in a substantial proportion of cases (Fig. 2 B), consistent with immune evasion status [28]. Cell lineage proportion analysis (CIBERSORTx) confirmed a significant infiltration of tumor-promoting immune cell populations in PNEN-B neoplasms, including CD4⁺, Tregs and CD8⁺ T cells, neutrophiles and undifferentiated M0 macrophages (Fig. 2 C). However, PD-L1 expression was not enhanced in this inflamed PNEN-B cluster (data not shown), speculating on different mechanisms of immunosuppression.

**Fig. 3:**
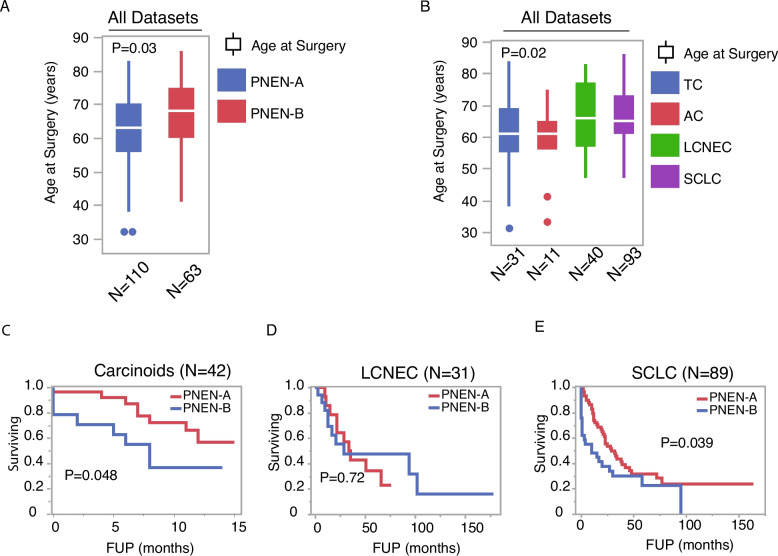
**A** Box plot analysis of age distribution in PNEN-A and PNEN-B NE tumors across all datasets analyzed. Statistical significance was assessed using Student’s t-test. **B** Box plot analysis of age distribution in NE tumor subtypes across all datasets analyzed. Statistical significance was assessed using ANOVA. **C-E** Kaplan-meier plots of patients with PNEN-A and B NE tumors of the indicated NE tumor types. Log-rank p-values are also shown. N, number of patients. FUP, follow-up

On the clinical side, elderly patients were more frequently in the PNEN-B cluster (*p* = 0.03; Fig. 3 A), consistent with the notion that NEC patients tend to be older (Fig. 3 B; *p* = 0.02). Lastly, worse overall survival was observed in PNEN-B carcinoids and SCLCs (*p* = 0.048 and *p* = 0.039, respectively; Fig. 3 C, E), but not in LCNECs (*p* = 0.72; Fig. 3 D), likely due to their recognized biological heterogeneity [21]. Multivariate Cox survival analysis confirmed the independent prognostic significance of the PNEN-B versus PNEN-A subgroup classification in carcinoids (HR, 5.86; *p* = 0.009), while a trend toward significance was observed in SCLC (HR, 1.59; *p* = 0.09), but not in LCNEC (Table 1).

**Table 1:** Multivariate survival Cox analysis of clusters and clinico-pathologic characteristics of different tumor subtypes

Carcinoid	**HR (95% CI)**	***P*** value^**a**^
**Age at Surgery**	1.13 (1.06–1.22)	0.0008*
**20-genes**^b^		
PNEN-B vs. -A	5.86 (1.54–22.2)	0.0094*
**Gender**^b^		
male vs. female	1.85 (0.39–8.92)	0.4406
**Smoking**^b^		
current vs. former	0.28 (0.03–2.76)	0.2734
current vs. never	1.29 (0.12–13.6)	0.8298
former vs. never	4.68 (1.10–19.9)	0.0365*
**LCNEC**		
**Age at Surgery**	1.03 (0.97–1.09)	0.3042
**20-genes**^b^		
PNEN-B vs. -A	0.75 (0.27–2.04)	0.5699
**Gender**^b^		
male vs. female	1.88 (0.59–5.95)	0.2828
**Smoking**^b^		
current vs. former	1.23 (0.44–3.41)	0.6881
**SCLC**		
**Age at Surgery**	1.02 (0.98–1.05)	0.3061
**20-genes**^b^		
PNEN-B vs. -A	1.59 (0.92–2.76)	0.0969
**Gender**^b^		
male vs. female	2.19 (1.16–4.16)	0.0157*
**Smoking**^b^		
current vs. former	1.52 (0.86–2.69)	0.147
current vs. never	1.18 (2.71–5.17)	0.8218
former vs. never	0.78 (0.17–3.53)	0.7447

^a^*P*-value, Wald test

^b^normal approximations used for ratio confidence limits effects

*P*-value < 0.05 indicates a strong evidence of an effect, and the variable is considered statistically significant

## Conclusion

In this study, we propose that a subset of lower-grade NETs, NE-high, embedded in the PNEN-B cluster, may undergo NE dedifferentiation and reprogramming, converging toward NE-low NECs, either SCLC or LCNEC, which are characterized by an enrichment of NSCLC-like features, including glandular, basal/squamous, or tuft cell markers as a result of remarkable cancer stem/progenitor cell plasticity. Because LCNECs exhibit a broad spectrum of NE and non-NE states (considerably more pronounced than in carcinoids and SCLCs, which are predominantly committed to a NE phenotype) [1], and given that LCNECs were also enriched within our PNEN-B cluster along with expression of MYC, RB1, YAP1, ASCL2, POU2F3, and basal cell markers, it can be hypothesized that they may represent intermediate and metastable hub states along this NE-high NET to NE-low SCLC transition trajectory. Notably, PNEN-A/B classification showed significant correlation with the currently proposed LCNECs molecular subtypes [5], thus further reinforcing our findings (Supplemental Table 1). Remarkably, PNEN-B tumors, despite dataset heterogeneity also due to alternative gene expression profiling technologies adopted (*i.e.,* microarray vs. RNA-seq), show the co-existence of different histological types formally recognized by the current WHO classification, *i.e.,* NETs, SCLCs and LCNECs, thereby suggesting a high-plasticity cell state. This plasticity in PNEN-B tumors, shifting toward adenocarcinoma, tuft cell carcinoma, or squamous cell carcinoma lineages with the expression of club/AT2 markers, POU2F3, or ΔNp63/p63/CK5, respectively, was also associated with increased biological aggressiveness (Table 2), despite similar demographic and clinicopathological trait distribution in NE-high PNEN-A tumors (Tables 3, 4, 5). Moreover, the PNEN-B cluster resulted independent in multivariate analysis for carcinoids and, marginally, SCLC (Table 1), but not in LCNEC, probably because of their inherent heterogeneity character realising a kind of molecularly metastable hub.

**Table 2:** Association analysis of clusters and clinico-pathologic characteristics of carcinoids and small cell lung carcinomas (data set from Asiedu et al.)

	**PNEN-A**	**PNEN-B**	***p***-value
N	28	26	-
**Age - average (SD)**	58 (±12.9)	64 (±10.8)	0.28^a^
**Gender**			
Male	11 (20.4%)	9 (16.7%)	0.78^b^
Female	17 (31.5%)	17 (31.5%)	
**Smoking Status**			
never smoker	14 (25.9%)	9 (16.7%)	0.0034^c^
former smoker	13 (24.07%)	7 (12.96%)	
current smoker	1 (1.85%)	10 (18.52%)	
**Pack-years**	36.2 (±32.5)	52.3 (±30.15)	0.09^a^
**Stage**			
I	21 (38.9%)	10 (18.5%)	0.0005^c^
II	6 (11.1%)	4 (7.4%)	
III-IV	1 (1.85%)	12 (22.2%)	
**Tissue Type**			
Typical Carcinoid	22 (41%)	9 (17%)	<0.0001^c^
Atypical Carcinoid	6 (11.1%)	5 (9.2%)	
SCLC	0 (0%)	12 (22%)	
**Overall Survival**			
dead	8 (14.81%)	20 (37.04%)	0.0005^b^
alive	20 (37.04%)	6 (11.1%)	

percentages could not add up to 100 due to rounding

^a^Wilcoxon test

^b^Fisher’s exact test

^c^Likelihood Ratio

**Table 3:** Association analysis of clusters and clinico-pathologic characteristics in small cell lung carcinomas (data set from George et al.)

	**PNEN-A**	**PNEN- B**	***p***-value
N	64	17	-
**Age - average (SD)**	64 (±8.82)	67.5 (±7.58)	0.17^a^
TMB - average (SD)	8 (±5.2)	9 (±5.84)	0.67^a^
**Gender**			
Male	45 (56%)	11 (14%)	0.77^b^
Female	19 (23%)	6 (7%)	
**Smoking Status**			
never smoker	2 (2.47%)	1 (1.23%)	0.39^c^
former smoker	29 (35.80%)	5 (6.17%)	
current smoker	30 (37.04%)	11 (13.58%)	
NA	3 (3.7%)	0 (0%)	
**Pack-years**	41 (±25.3)	49 (±23.3)	0.22^a^
**Stage**			
I	23 (28%)	11 (14%)	0.24^c^
II	13 (16%)	2 (2%)	
III-IV	26 (32%)	4 (5%)	
NA	2 (2%)	0 (0%)	
**Overall Survival**			
dead	38 (49.3%)	10 (13%)	0.78^b^
alive	22 (28.6%)	7 (9.1%)	

percentages could not add up to 100 due to rounding

^a^Wilcoxon test

^b^Fisher’s exact test

^c^Likelihood Ratio

**Table 4:** Association analysis of clusters and clinico-pathologic characteristics in large cell neuroendocrine carcinomas (data set from George et al.)

	**PNEN-A**	**PNEN-B**	***p***-value
N	19	21	-
**Age - average (SD)**	63 (±10.11)	67.5 (±9.5)	0.22^a^
**Gender**			
Male	11 (27.5%)	18 (45%)	0.077^b^
Female	8 (20%)	3 (7%)	
**Smoking Status**			
former smoker	6 (15.0%)	9 (22.5%)	0.6^c^
current smoker	12 (30.0%)	10 (25.0%)	
NA	1 (2.5%)	2 (5%)	
**Stage**			0.97^c^
I	8 (20.0%)	9 (22.5%)	
II	4 (10.0%)	5 (12.5%)	
III-IV	6 (15.0%)	6 (15.0%)	
**Overall Survival**			
Dead	10 (25.0%)	10 (25.0%)	0.7^b^
Alive	4 (10.0%)	7 (17.5%)	
NA	5 (12.5%)	4 (10.0%)	

percentages could not add up to 100 due to rounding

^a^Wilcoxon test

^b^Fisher’s exact test

^c^Likelihood Ratio

**Table 5:** Association analysis of clusters and clinico-pathologic characteristics of carcinoids (data set from Laddha et al.)

	**PNEN-A**	**PNEN-B**	***p***-value
N	15	15	-
**Age - average (SD)**	50 (±16.2)	65.7 (±13.29)	0.01^a^
**Gender**			
Male	7 (23.3%)	2 (6.7%)	0.11^b^
Female	8 (26.7%)	13 (43.3%)	
**Tissue Type**			
Typical Carcinoid	10 (33.3%)	7 (23.3%)	0.46^b^
Atypical Carcinoid	5 (11.1%)	8 (26.7%)	
**Lymphnodal Met**			
Yes	0 (0%)	4 (13.3%)	0.1^b^
No	15 (50%)	11 (36.7%)	

percentages could not add up to 100 due to rounding

^a^Wilcoxon test

^b^Fisher’s exact test

Mechanistically, both PNEN-A and PNEN-B tumors are characterized by abundant inflammatory infiltrates and preserved MHC/HLA expression. However, only PNEN-B tumors tend to develop a dominant immunosuppressive milieu, likely due to epigenetic mechanisms, together with EMT mechanisms and the acquisition of stem cell-like properties, ultimately leading to functional exhaustion of infiltrating T cells, enrichment of other tumor-promoting immune cells, and aggressive behavior (Figure 2 B-C). Within this framework, PNEN-B tumors differ from PNEN-A tumors by different adaptive evolutionary mechanisms promoting NE downregulation and lineage reprogramming. This processes involve activation of NOTCH1 [29, 30], YAP1 [29, 30], MYC, RB1 [31], components of the SWI/SNF chromatin-remodeling complex, and fascin overexpression (Fig. 1 A and Supplemental Figure 1 A) [32]. Such changes are probably mediated by TIME interactions, and resemble those observed in NE-low SCLC-like/type II LCNECs and LCNEC-like SCLC [5] or in therapy-resistant non-NE SCLC variant subtypes derived by Gazdar in chemotherapy-treated human cancers [33, 34], which are similarly enriched in immunosuppression, EMT mechanisms and stem cell-like features. The extensive TIME remodeling in PNEN-B tumors may shift from an MHC/HLA-proficient, antitumor context to an MHC/HLA-blocked immunosuppressive niche (Supplemental Figure 2 and Fig. 3), potentially enabling responsiveness to PD-L1 inhibitor-independent immunotherapeutic strategies [20], and ultimately favoring development of aggressive NSCLC-like tumors. Hormones associated with NE cells, specifically calcitonin (CALCA), proopiomelanocortin (POMC), and gastrin-releasing peptide (GRP), may contribute to the establishment of such immunosuppressive environment, which is in line with our observation of chronic IFN signaling facilitating immune evasion (Supplemental Figure 1 A). This study is consistent with previous findings we obtained using a different gene signature, in which we identified a cluster of carcinoids, designated CL2, which shared molecular features of high-grade NECs along with an inflamed but immunosuppressive landscape, suggesting the existence of non-random mechanisms underlying the transition of certain carcinoid subsets to NECs [30]. The coexistence of different histologic subtypes in PNEC-B tumors supports the concept of functionally “metastable” lesions, in which NE differentiation is epigenetically regulated and reprogrammed toward NE-low NECs, also with NSCLC-like features [34–36]. These ASCL1-low/ASCL2-high/NOTCH-high/YAP1-high/RB1-high PNEN-B tumors of the present study, along with the previous CL2 tumors [30], are complementary to the recently defined supra-carcinoids arising as NE-dedifferentiated microregional subclones in conventional carcinoids, which exhibit either immunosuppressed or immune-desert landscape (the latter enriched in ASCL1), are biologically aggressive, are composed of undifferentiated progenitor cells of the lower airways (the so-called, LAP cells), and share molecular features with high-grade NECs **(**Sexton-Oates A et al., *Mol Cancer* 2026, accepted for publication, 10.1186/s12943-026-02721-7). Our PNEN-B tumors, even though share phenotypes of NET, NEC and NSCLC, are nevertheless different from pulmonary or extrapulmonary adenocarcinomas transdifferentiating to small-cell NE phenotype [37, 38], or from chromotripsis-mediated SCLC developing from pre-existing carcinoids [38], both of which eventually exhibit ASCL1-high and DLL3-high phenotype, along with desert-like immune microenvironment. In other words, taken together, these studies suggest the existence of at least two distinct routes toward high-grade NEC phenotype, either inflamed or immune-desert. On the one hand, there would be an inflamed NET-derived trajectory encompassing NE dedifferentiation and NSCLC-like reprogramming as previously observed in the cluster CL2 carcinoids [30, 39] and, presently, in PNEN-B carcinoids and in a subset of highly inflamed/YAP1-enriched supra-carcinoids, transcriptomically close to LCNEC (Sexton-Oates A et al., *Mol Cancer* 2026, accepted for publication, 10.1186/s12943-026-02721-7). On the other hand, there would be an immune-desert, ASCL1-high, DLL3-positive trajectories represented by chromotripsis-mediated atypical SCLC deriving from pre-existing carcinoids [38], or the plastic small cell NE lineage state described by Balanis NG et al. and widespread across many epithelial and non-epithelial cancers [37]. Intriguingly, PNEN-B carcinoids were enriched for NE-low programs (MYC/NOTCH/YAP1) but frequently retained RB1 and, unlike canonical NE-low SCLC, often co-expressed ASCL1 and NEUROD1, resembling supra-carcinoids with preserved neuroendocrine features. Across independent cohorts, these findings suggest that PNEN-B represents an evolutionary or metastable state rather than a discrete molecular subtype. Such tumors may undergo lineage plasticity toward stemness-enriched, therapy-resistant phenotypes through activation of the NOTCH/MYC–YAP1 axis [40, 41], while RB1-retaining SCLCs have also been associated with greater biological aggressiveness [31, 42, 43].

Although the proposed evolutionary mechanisms are inferred from the observation of molecular sharing within specific clusters, without providing a direct transformation of carcinoid cells into NECs on the basis of *in vitro* and/or *in vivo* experiments, our findings yielded consistent across distinct and using an *a priori* selected, molecular signature, and were somewhat independent of the WHO classification tumor types. Together with the evidence from independent studies underlying the inherent molecular heterogeneity of pulmonary NENs [5, 44, 45], our findings suggest that multiple molecular pathways are likely to underlie the plasticity of cancer stem/progenitor cells, thereby promoting a wide array of possible evolutionary trajectories in lung cancer morphogenesis, also suggesting potential therapeutical vulnerabilities (e.g., SMARCA4/2 inhibitors in POU2F3-enriched SCLC; ClinicalTrials.gov ID #NCT07551635).

## Supplementary Information

Supplementary Material 1.

Supplementary Material 2.

Supplementary Material 3.

Supplementary Material 4.

Supplementary Material 5.

## Data Availability

No datasets were generated or analysed during the current study.

## Abbreviations

AC: atypical carcinoid
LCNEC: large cell neuroendocrine carcinoma
NE: neuroendocrine
NEC: neuroendocrine carcinoma
NEN: neuroendocrine neoplasm
NET: neuroendocrine tumors
NSCLC: non-small cell carcinoma
PNEN-A: pulmonary neuroendocrine neoplasm-cluster A
PNEN-B: pulmonary neuroendocrine neoplasm-cluster B
SCLC: small cell lung carcinoma
TC: typical carcinoid
TIME: tumor immune microenvironment
TKI: tyrosyne kinase inhibitor
