# Supplementary material for: Transcriptional evidence of neuroendocrine cell plasticity beyond histological boundaries in lung neuroendocrine neoplasms: an in-silico analysis suggesting a progression model
Source: J Exp Clin Cancer Res. 2026 Jul 28;45:168. doi: 10.1186/s13046-026-03790-8 (PMC13418738; doi:10.1186/s13046-026-03790-8)
Supplement: Supplementary file 1 — Supplementary Material 1. [file 13046_2026_3790_MOESM1_ESM.docx]

**SUPPLEMENTAL METHODS**

This study comprised a primary treatment-naïve cohort of 81 SCLCs and 40 lung LCNECs, which had previously been published by George et al. in 2015 (FPKM data level; [1]) and 2018 (FPKM data level; [2]). A third cohort of 54 available out of 63 lung NENs comprising 39 TCs, 12 ACs carcinoids, 12 SCLCs along with 26 corresponding normal lung tissues [3], and a fourth independent cohort of 30 lung carcinoids (17 TCs and 13 ACs) [4] also entered the study.

*Study design.* This is an in-silico study aimed to investigate the relationship existing between NE differentiation and tumor microenvironment characteristics, with insights into the epigenetic changes involved in such a connection. Previously, we provided evidence by unsupervised clustering of transcriptomic data that there was an aggressive subset of lung carcinoids sharing molecular features of high-grade neuroendocrine neoplasms, where an immunosuppressive microenvironment could play some role in their development [5]. The small number of available carcinoids and the presence of SCLCs only prevented to draw conclusive remarks about this biological relationship. Therefore, in this study, we further expanded our lung NEN series to explore the relationship of NE downregulation and immune dysregulation by exploiting three additional independent datasets, totaling 205 lung NENs. These datasets span LCNECs, SCLCs, and carcinoids and were used to test their ability to highlight and discriminate this phenomenon.

*Bioinformatics and statistics.* Normalized gene expression data were downloaded from Gene Expression Omnibus (GEO) database (accession #: GSE108055; and, GSE118131) or from Supplementary Data [1, 2] and were applied to: i) hierarchical clustering analysis using uncentered correlation and centroid linkage using Cluster 3.0 (<http://bonsai.hgc.jp/~mdehoon/software/cluster/manual/>) and Java TreeView 1.2 (http://jtreeview.sourceforge.net) [6, 7]; ii) Gene-Set Enrichment Analysis (GSEA, <https://www.gsea-msigdb.org/gsea/index.jsp>) were performed using Hallmark gene sets with 1000 random permutations of data to calculated statistics of enrichment; and iii) CIBERSORTx analysis (<https://cibersortx.stanford.edu/>) using the LM22 signatures representing 22 immune cell types [8, 9]. JMP 19 (JMP®, Version 19. SAS Institute Inc., Cary, NC, 1989–2019) was used for all statistical analyses and relative plots. All *p*-values were two-sided and p-values *<*0.05 were considered as significant. Venn diagram was produced using “Venn” webtool (<https://bioinformatics.psb.ugent.be/webtools/Venn/>).

*20-gene signature selection criteria.*  The 20-gene signature we manually curated encompass key mechanisms involved in: i) NE differentiation and core oncogenic pathways, including MYC, NOTCH1, TP53, RB1, SFN, and members of the SWI/SNF (BAF) chromatin-remodeling complex (ARID1A, ARID1B, ARID2, SMARCA2, SMARCA4, SMARCB1); ii) canonical NE markers, such as chromogranin A/B (CHGA, CHGB), synaptophysin (SYN1), and insulinoma-associated protein 1 (INSM1); iii) lineage-defining transcription factors (TFs), including ASCL1, ASCL2, NEUROD1, POU2F3, and YAP1. In particular, this 20-gene signature was a priori selected on the basis of established molecular knowledge of pulmonary NENs and was not derived from, nor optimized on, the present study cohort to maximize classification performance. The rationale for selecting this signature was to provide a biologically interpretable and parsimonious representation of these key events driving lung NEN evolutionary biology, thereby minimizing the risk of overfitting and ensuring mechanistic interpretability.

**Supplemental Table and Figure legends**

**Supplemental Table 1.** Contingency analysis of LCNECs molecular subtypes in the PNEN-A/B classification. P-values were calculated by Likelihood Ratio-test.

**Supplemental Figure 1.** **A)** Box-plot analysis of the expression profile of the indicated genes. Y-axes, normalized gene expression. Statistical significance was assessed using Wilcoxon two-sample test. **B)** Box-plot analysis of the expression profiles of the indicated genes. ΔNp63 expression was available only in the George et al. SCLC dataset and was derived from gene expression data corresponding to transcript variant 4 of the TP63 gene (NM_001114980), also known as ΔNp63α (deltaNp63alpha, deltaN-alpha, p51delNalpha, CUSP, and p73H). Statistical significance was assessed using the Wilcoxon two-sample test. For the other two datasets shown, only gene-level expression data for TP63 were available. In the Asiedu et al. dataset, TP63, KRT5 and POU2F3 expression data were not available.

**Supplemental Figure 2.** Oneway analysis of Human Leukocyte Antigen (HLA) genes expression in PNEN-A (A) and in PNEN-B (B) clusters of SCLC (George et al.) samples. No significant differences were found.

**Supplemental Figure 3**. Oneway analysis of Human Leukocyte Antigen (HLA) genes expression in PNEN-A (A) and in PNEN-B (B) clusters of carcinoids samples. Increase of HLA expression (p<0.05, Wilcoxon test) was documented in the PNEN-B according to the Asiedu’s but not Laddha’s series of carcinoids.

**References (separately for Supplemental Methods)**

1. George J, Lim JS, Jang SJ, Cun Y, Ozretic L, Kong G, et al. Comprehensive genomic profiles of small cell lung cancer. Nature. 2015;524(7563):47-53.

2. George J, Walter V, Peifer M, Alexandrov LB, Seidel D, Leenders F, et al. Integrative genomic profiling of large-cell neuroendocrine carcinomas reveals distinct subtypes of high-grade neuroendocrine lung tumors. Nat Commun. 2018;9(1):1048.

3. Asiedu MK, Thomas CF, Jr., Dong J, Schulte SC, Khadka P, Sun Z, et al. Pathways Impacted by Genomic Alterations in Pulmonary Carcinoid Tumors. Clin Cancer Res. 2018;24(7):1691-704.

4. Laddha SV, da Silva EM, Robzyk K, Untch BR, Ke H, Rekhtman N, et al. Integrative Genomic Characterization Identifies Molecular Subtypes of Lung Carcinoids. Cancer Res. 2019;79(17):4339-47.

5. Pelosi G, Melocchi V, Dama E, Hofman P, De Luca M, Albini A, et al. An in-silico analysis reveals further evidence of an aggressive subset of lung carcinoids sharing molecular features of high-grade neuroendocrine neoplasms. Exp Mol Pathol. 2024;135:104882.

6. Pelosi G, Bianchi F, Dama E, Metovic J, Barella M, Sonzogni A, et al. A Subset of Large Cell Neuroendocrine Carcinomas in the Gastroenteropancreatic Tract May Evolve from Pre-existing Well-Differentiated Neuroendocrine Tumors. Endocr Pathol. 2021;32(3):396-407.

7. Pelosi G, Bianchi F, Dama E, Simbolo M, Mafficini A, Sonzogni A, et al. Most high-grade neuroendocrine tumours of the lung are likely to secondarily develop from pre-existing carcinoids: innovative findings skipping the current pathogenesis paradigm. Virchows Arch. 2018;472(4):567-77.

8. Chen B, Khodadoust MS, Liu CL, Newman AM, Alizadeh AA. Profiling Tumor Infiltrating Immune Cells with CIBERSORT. Methods Mol Biol. 2018;1711:243-59.

9. Newman AM, Steen CB, Liu CL, Gentles AJ, Chaudhuri AA, Scherer F, et al. Determining cell type abundance and expression from bulk tissues with digital cytometry. Nat Biotechnol. 2019;37(7):773-82.
