# Supplementary figures and images for: Transcriptional evidence of neuroendocrine cell plasticity beyond histological boundaries in lung neuroendocrine neoplasms: an in-silico analysis suggesting a progression model
Source: J Exp Clin Cancer Res. 2026 Jul 28;45:168. doi: 10.1186/s13046-026-03790-8 (PMC13418738; doi:10.1186/s13046-026-03790-8)

A

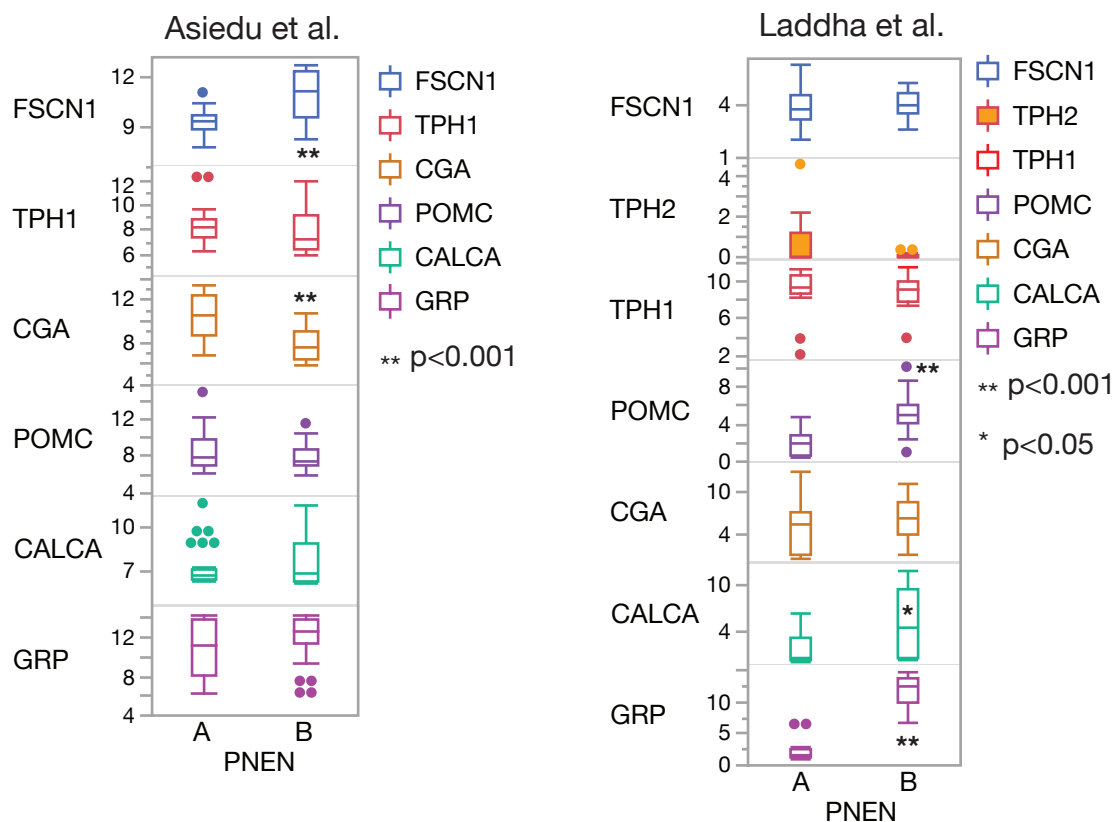

B

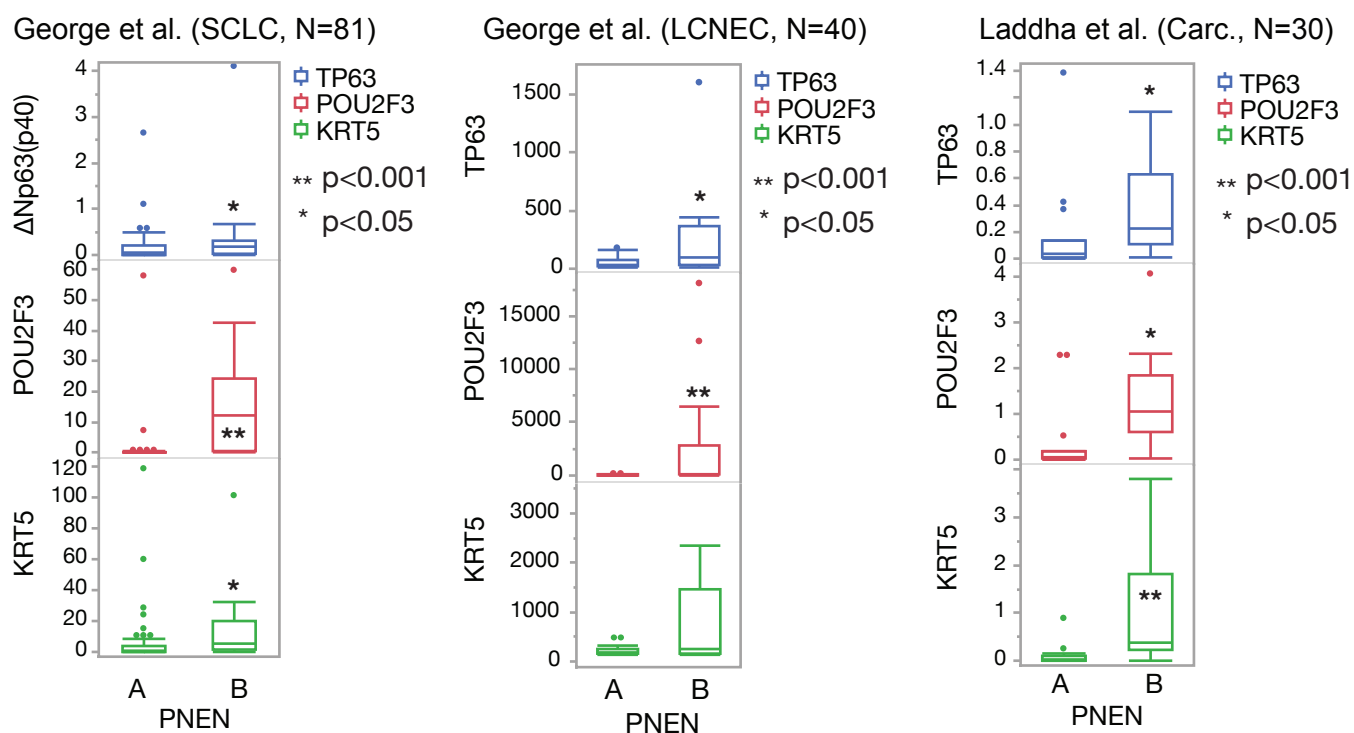

Figure S1

Supplement: Supplementary file 2 — Supplementary Material 2. [file 13046_2026_3790_MOESM2_ESM.pdf]

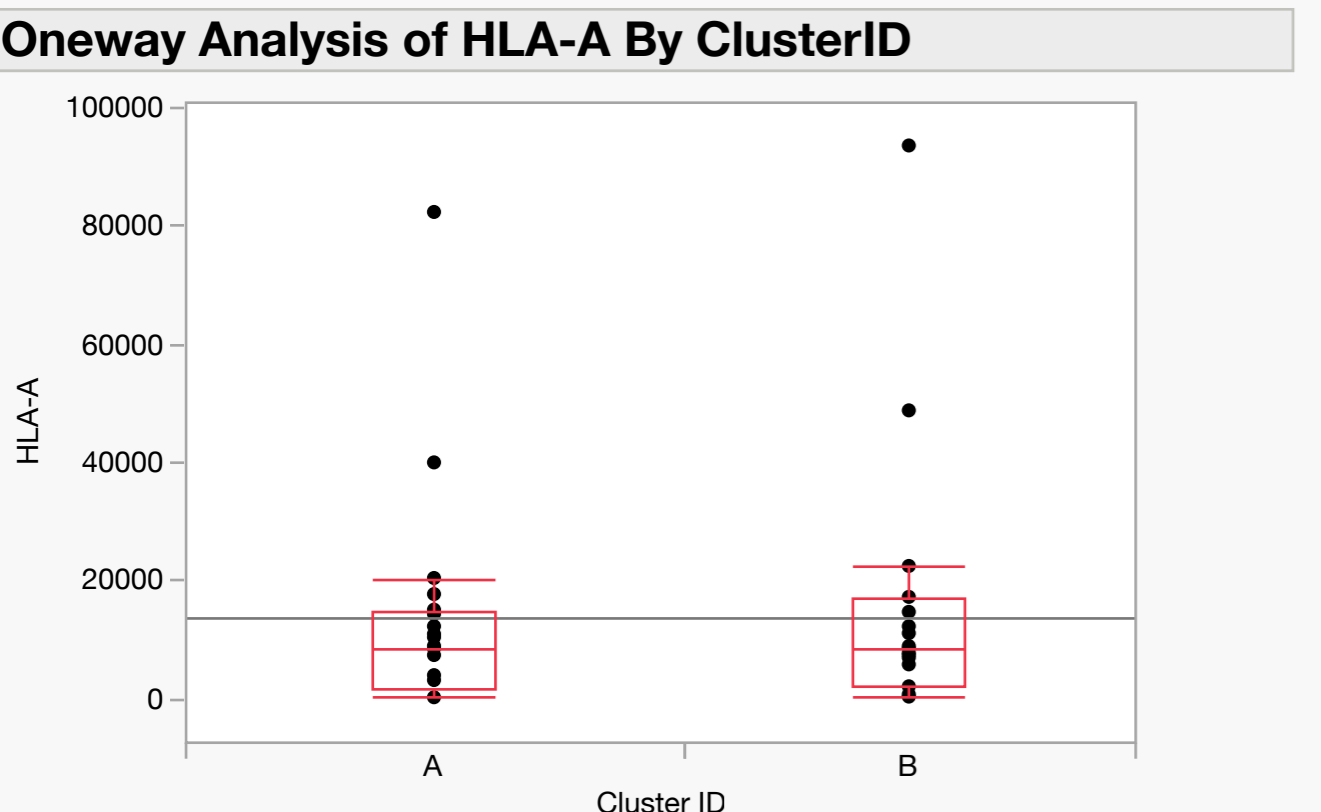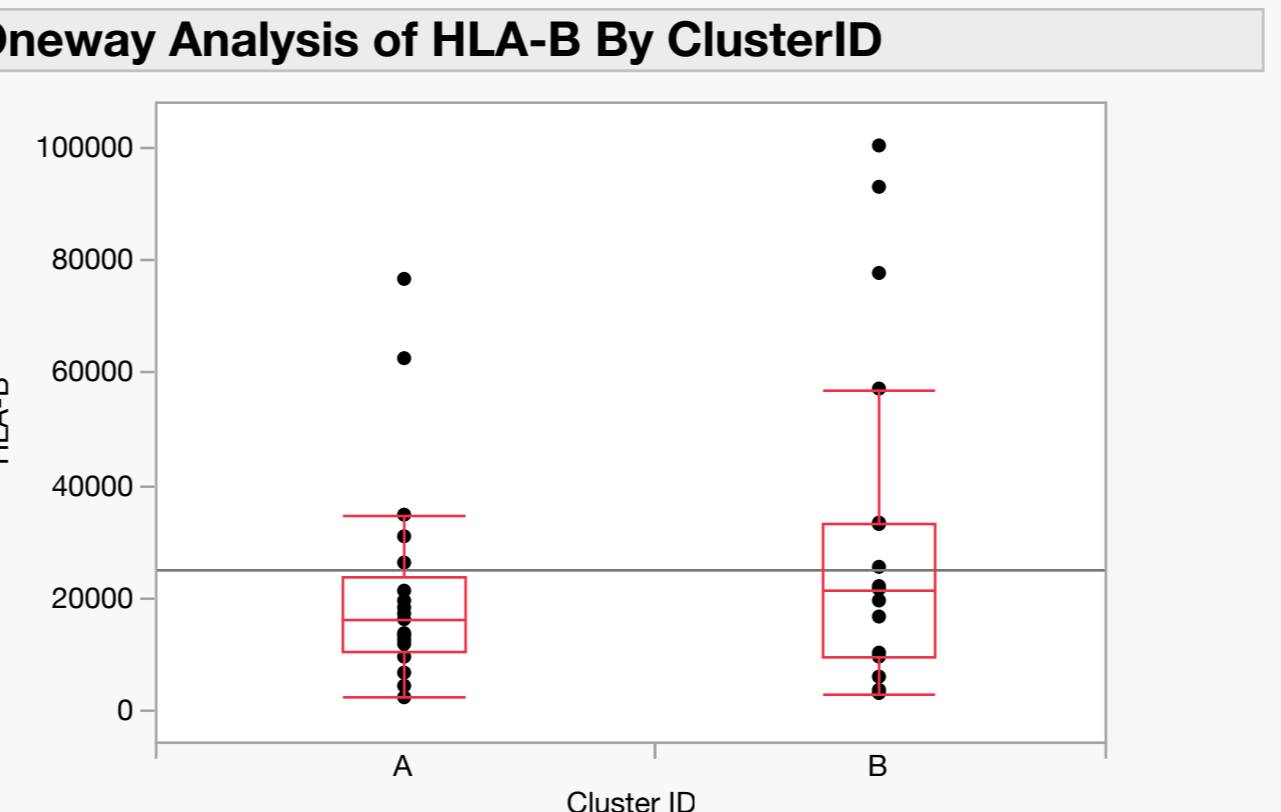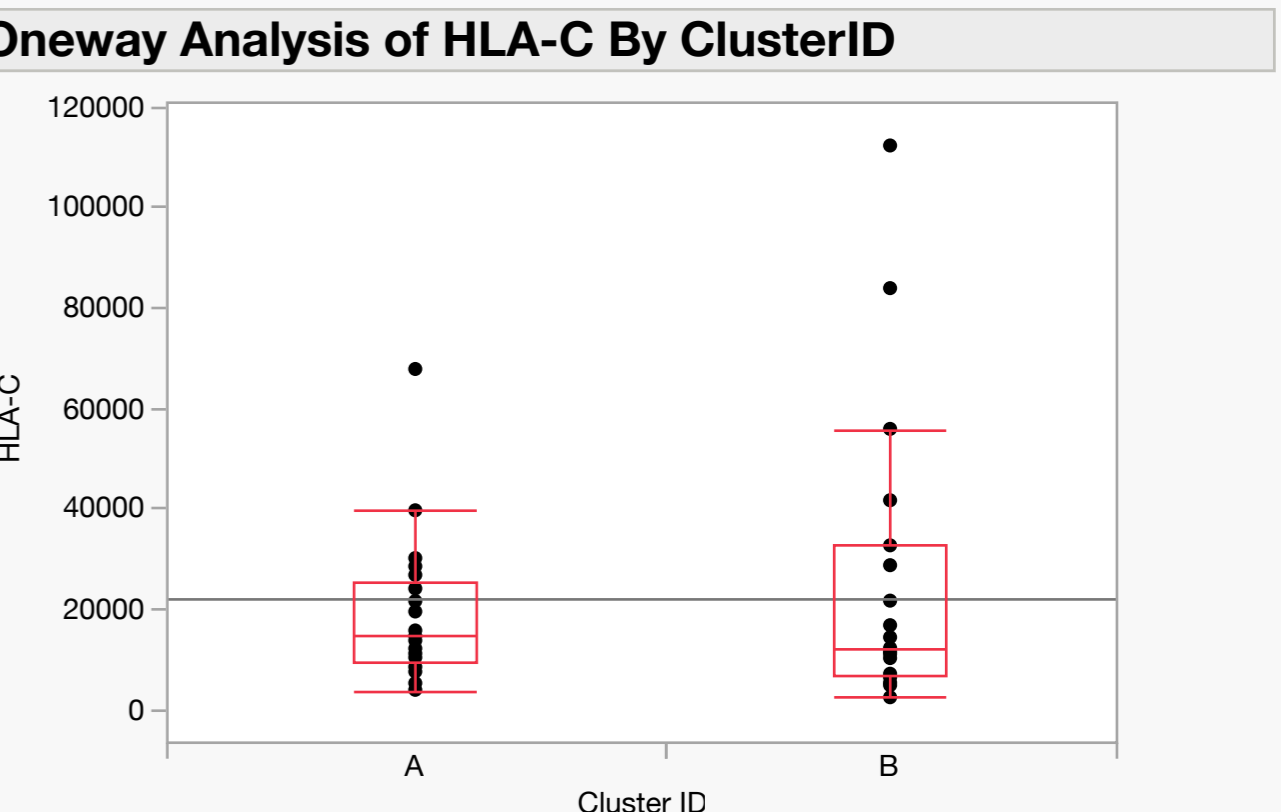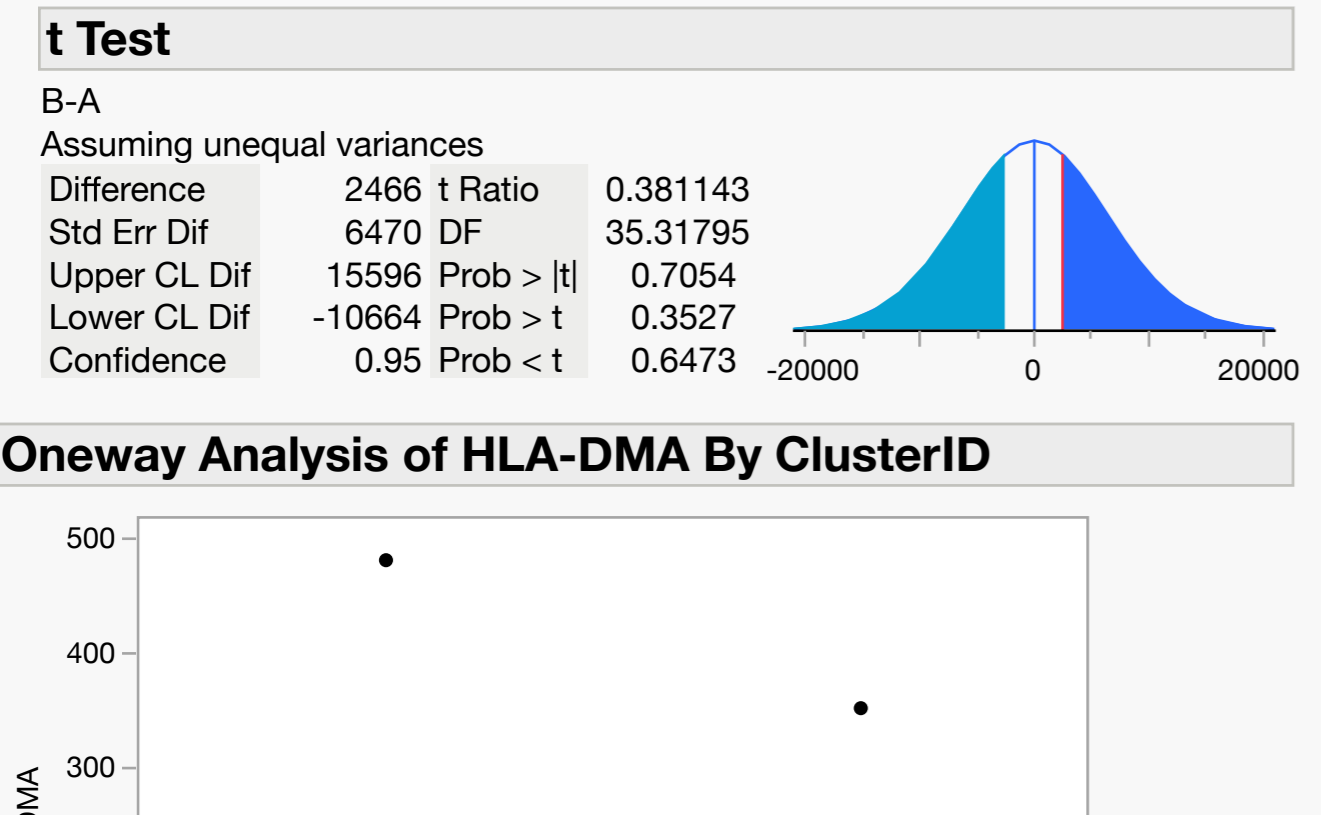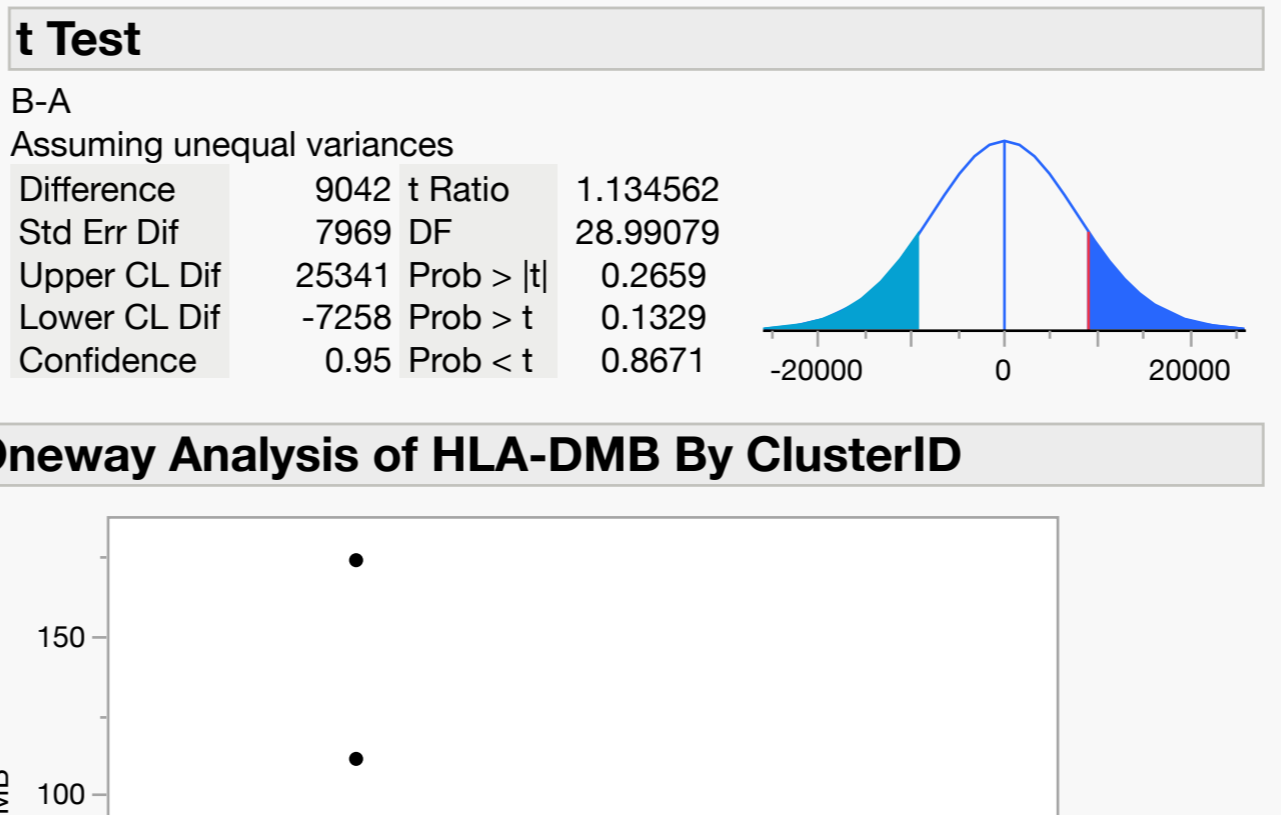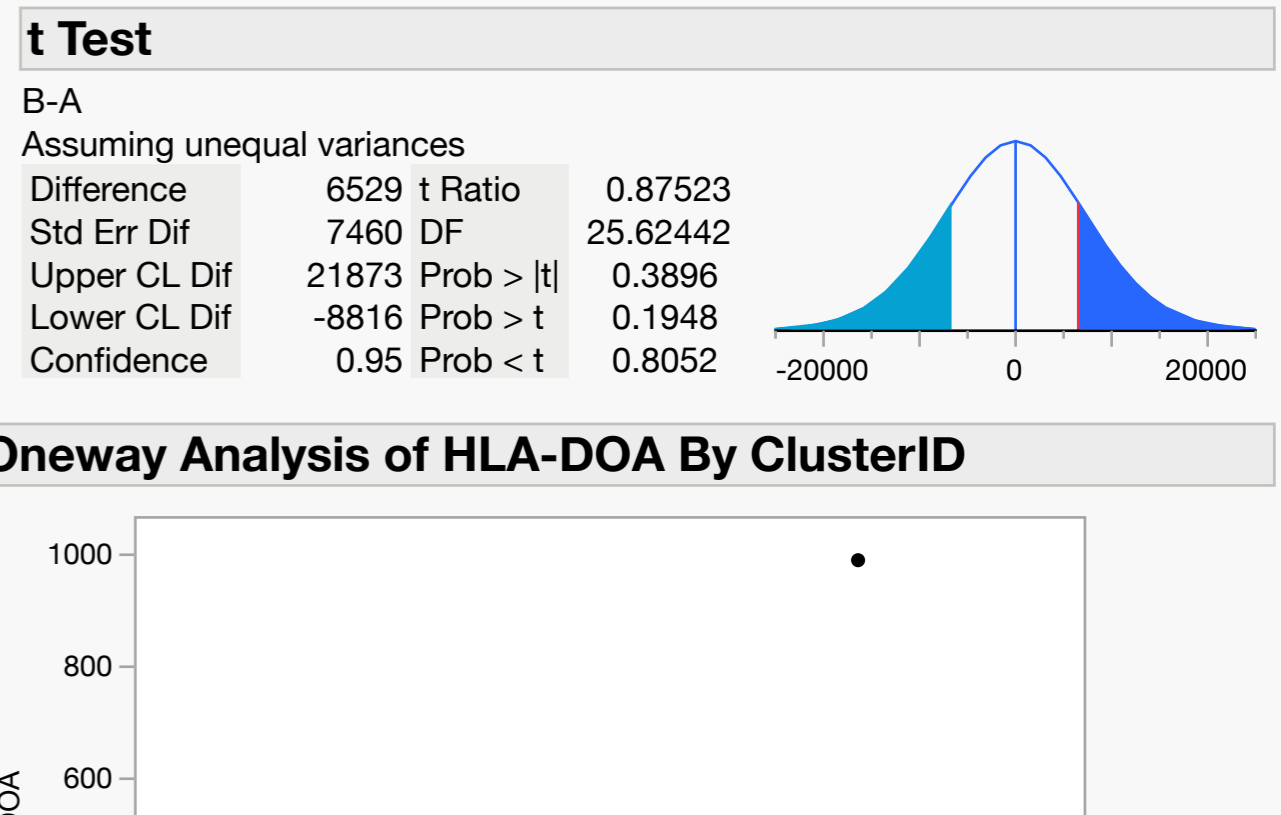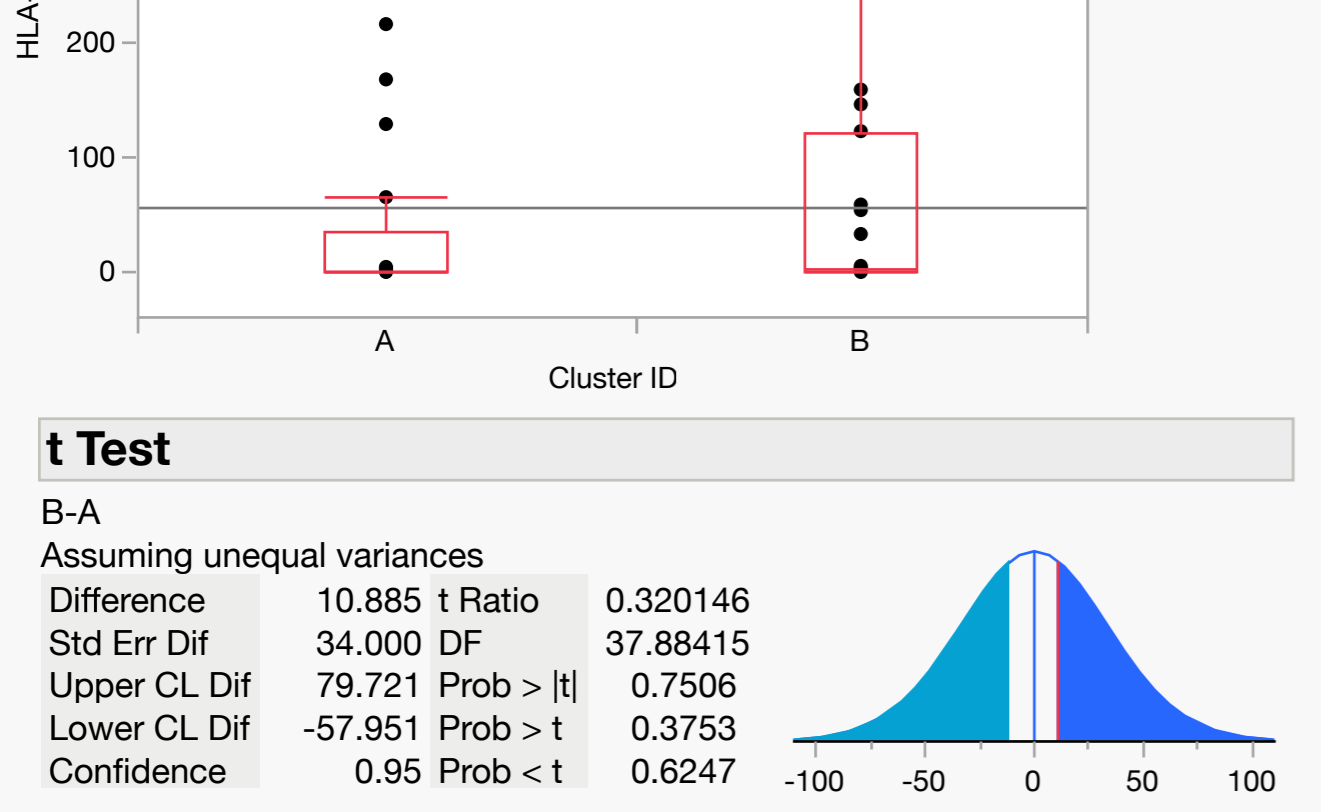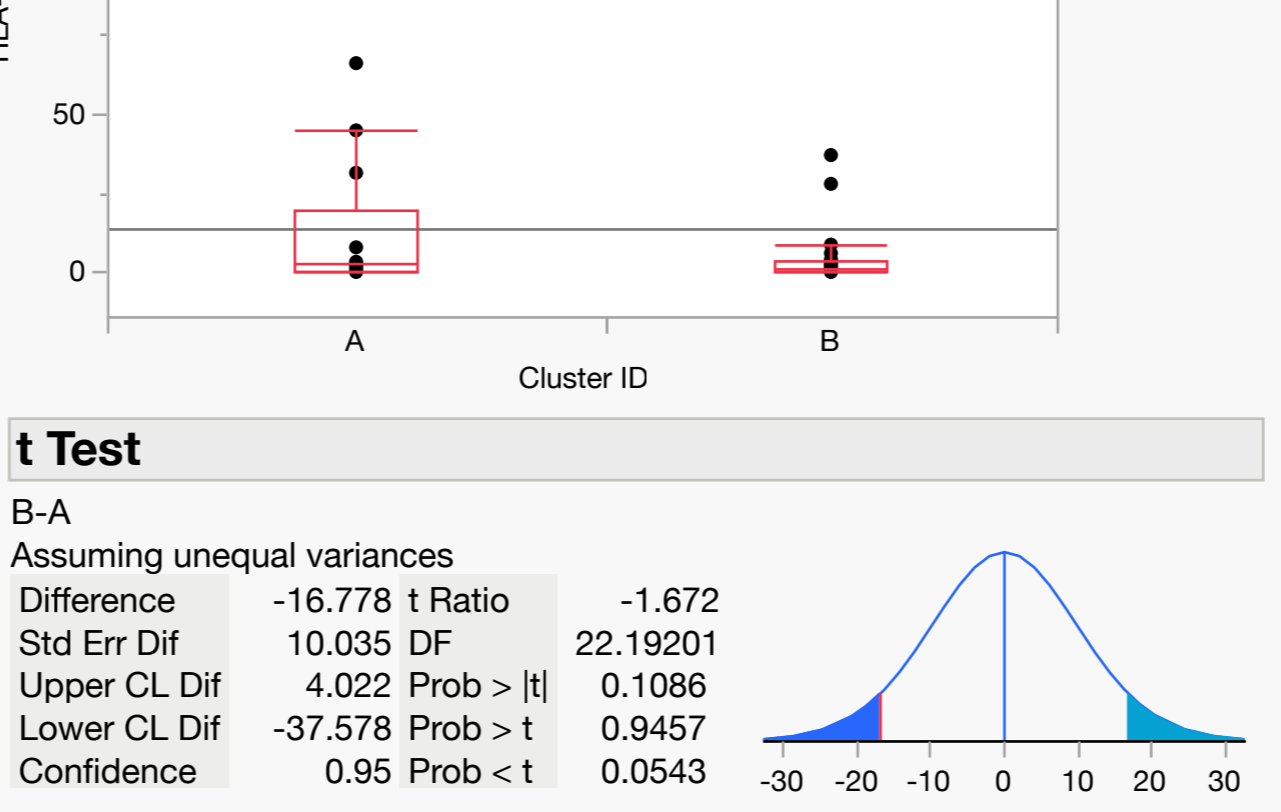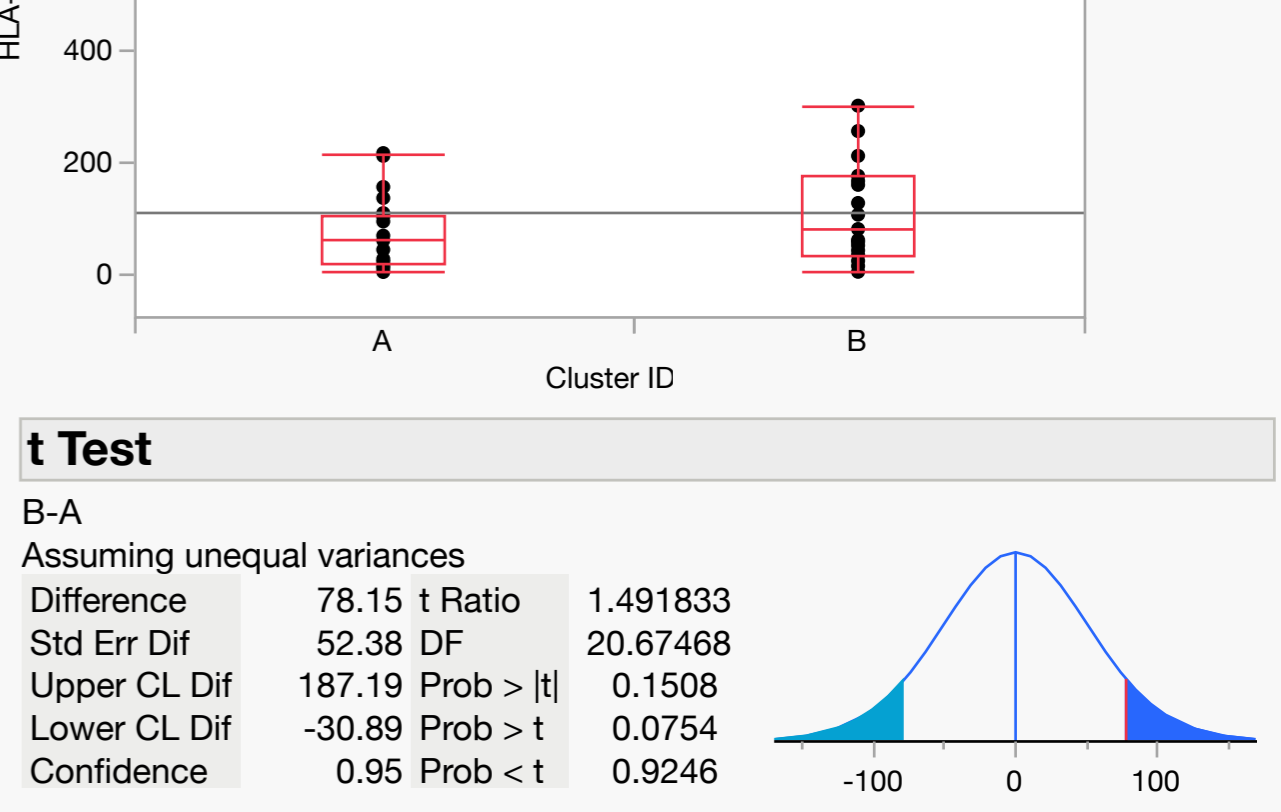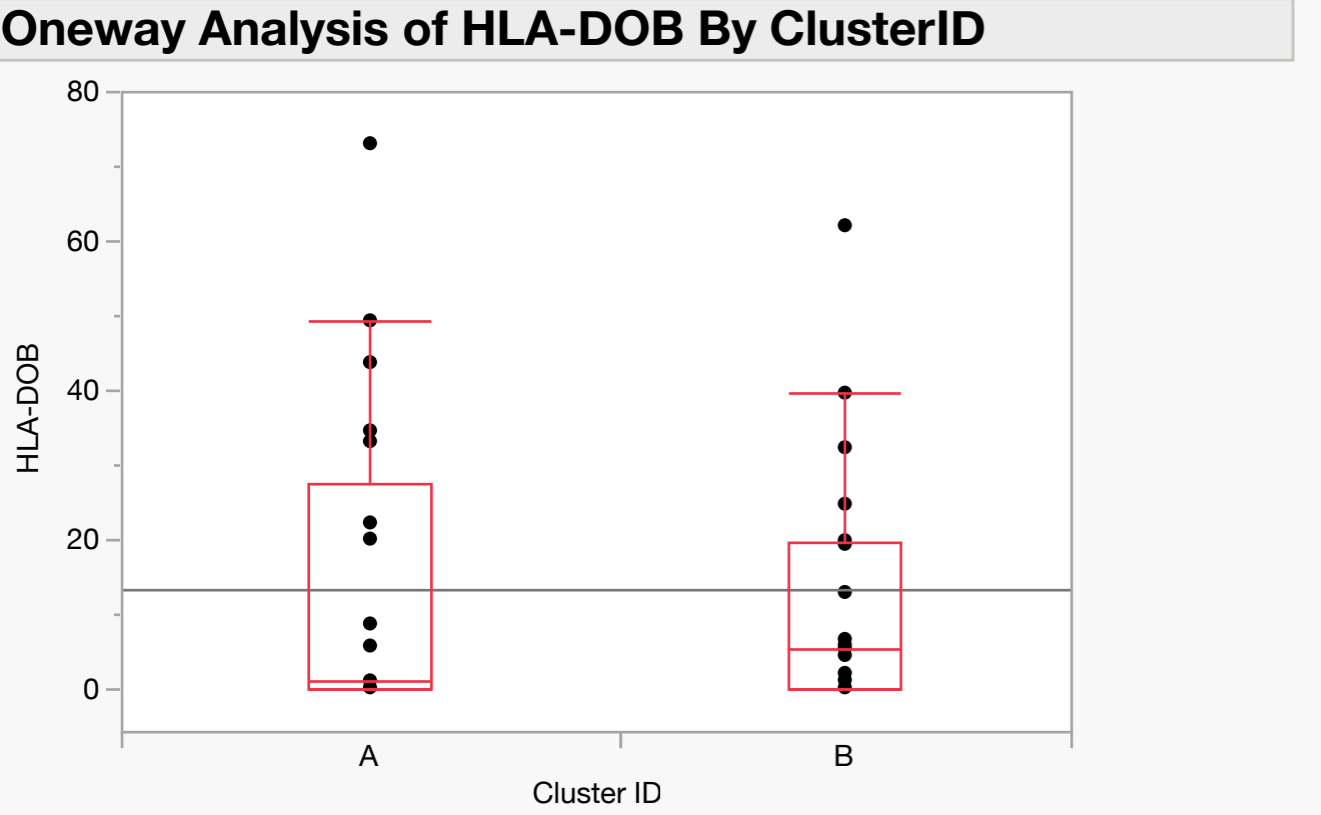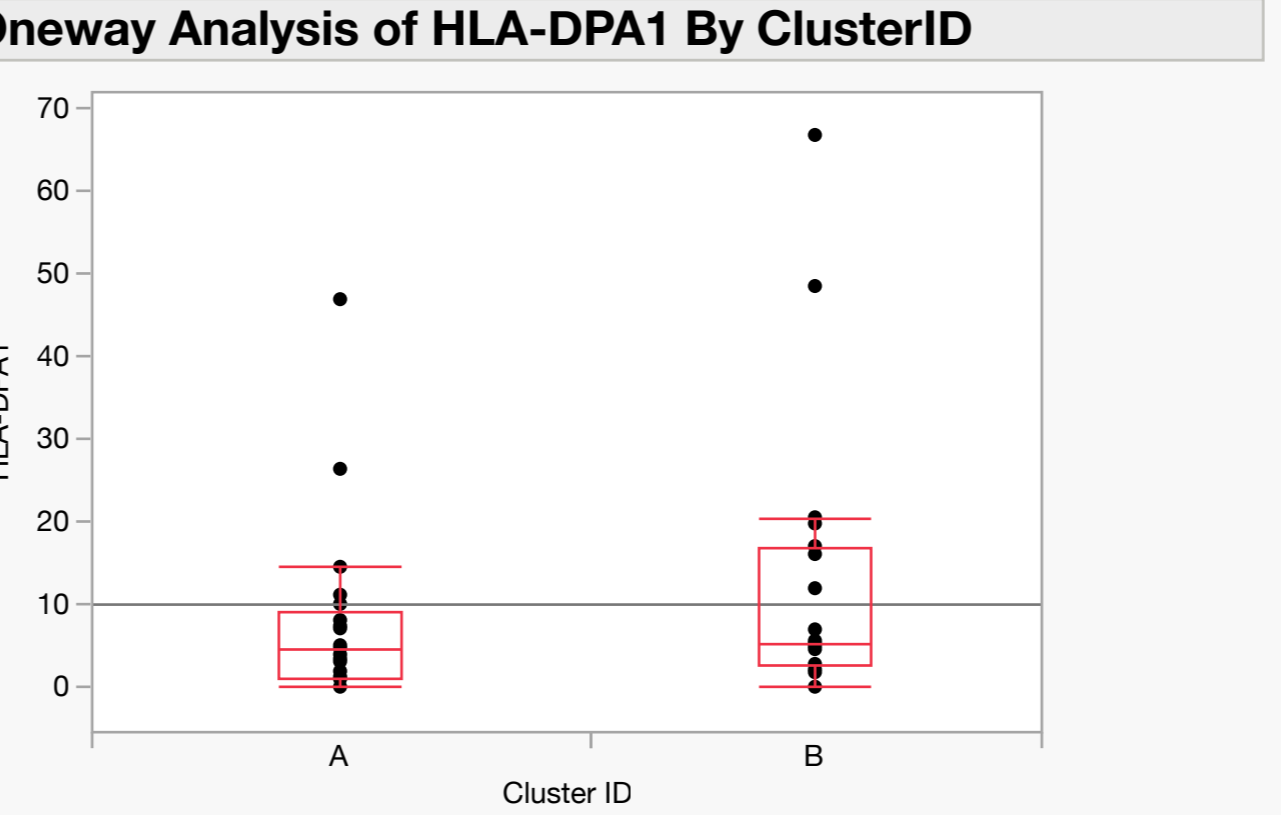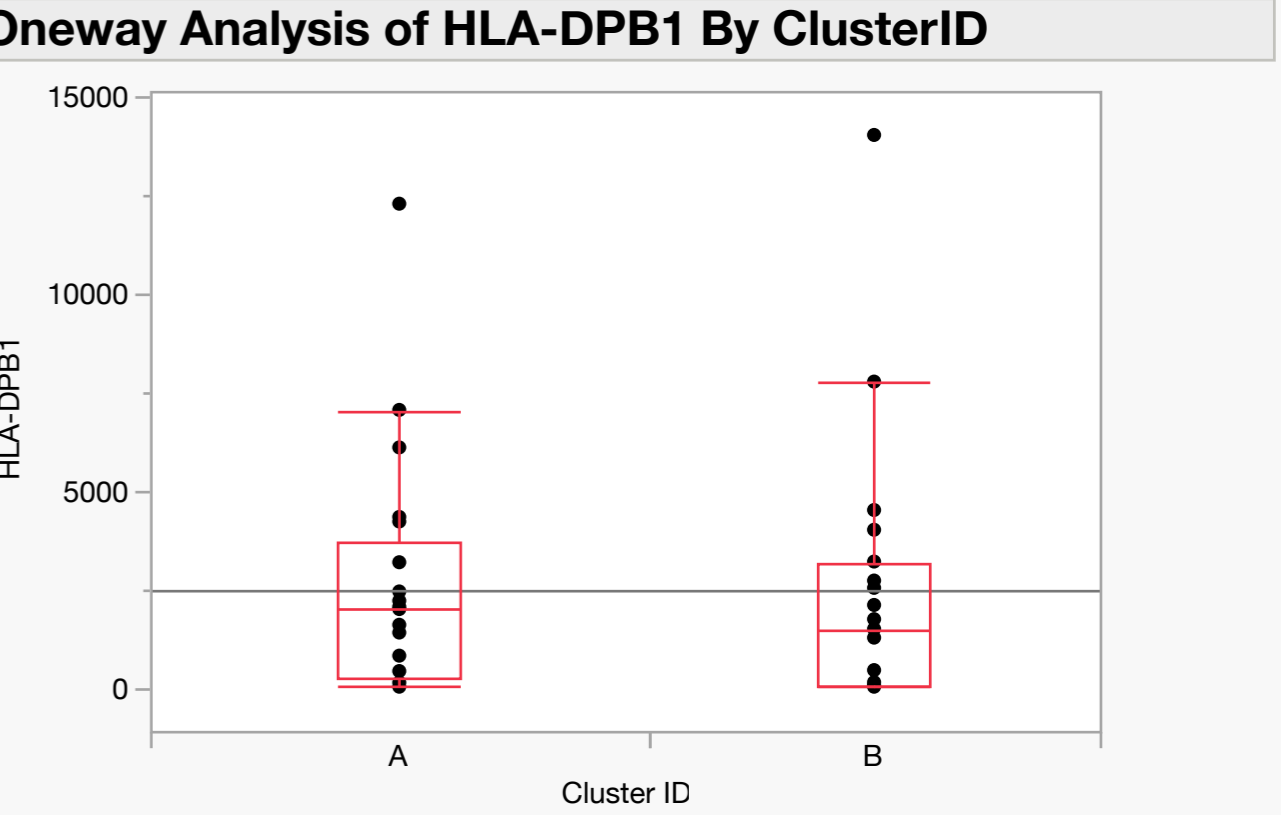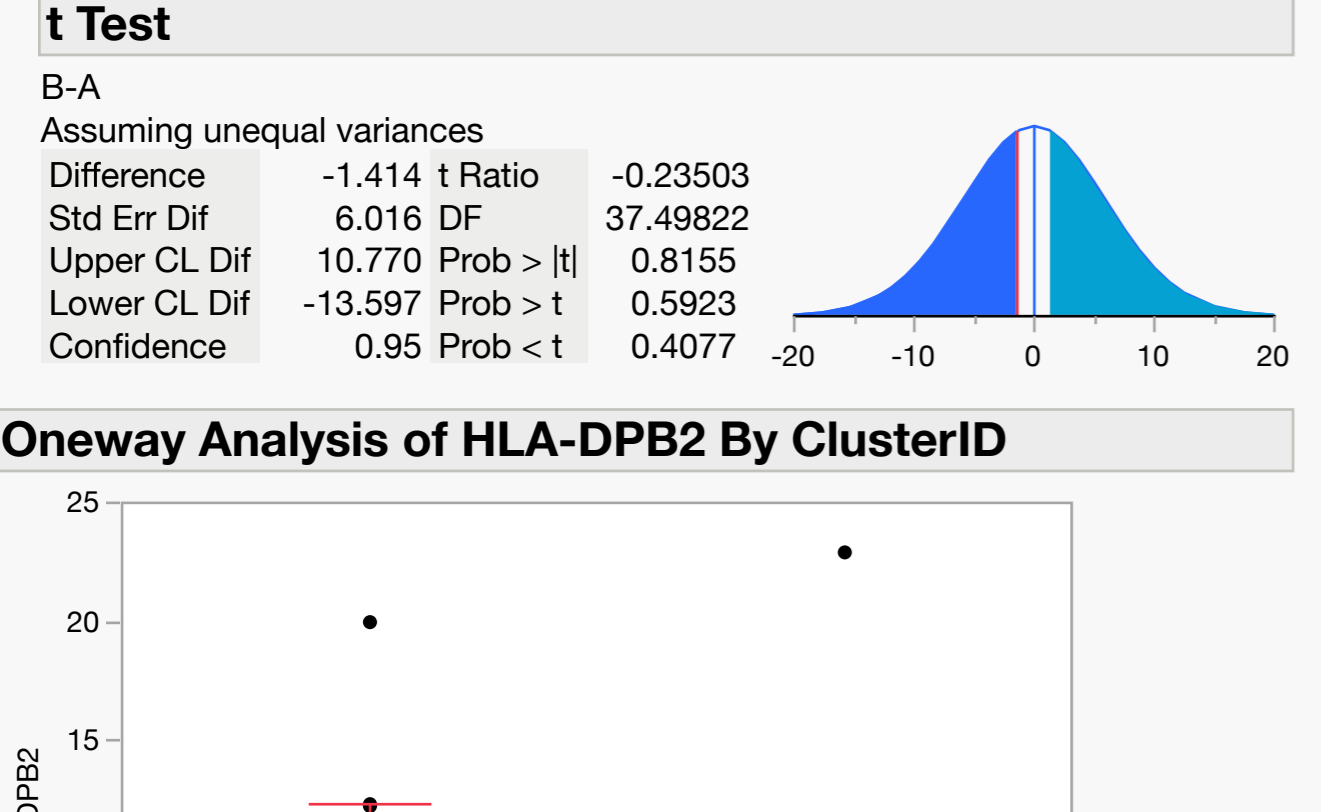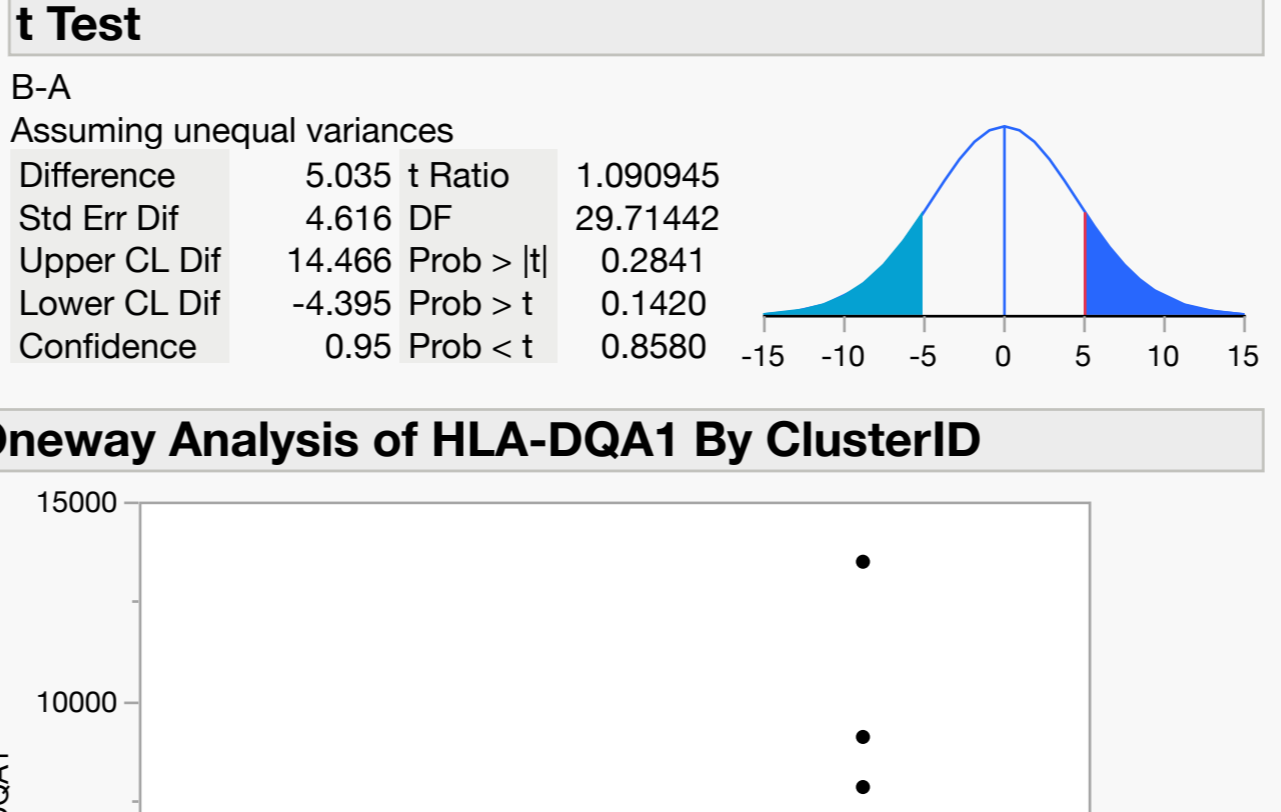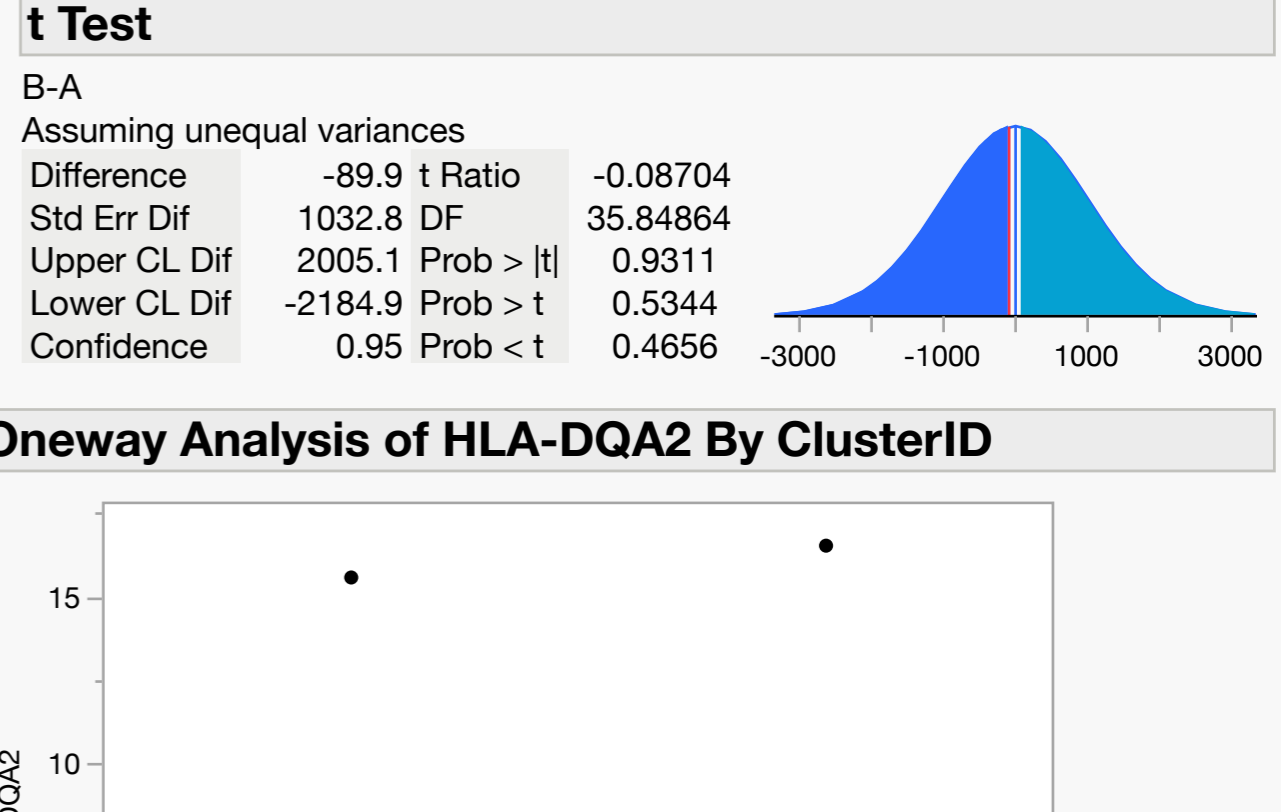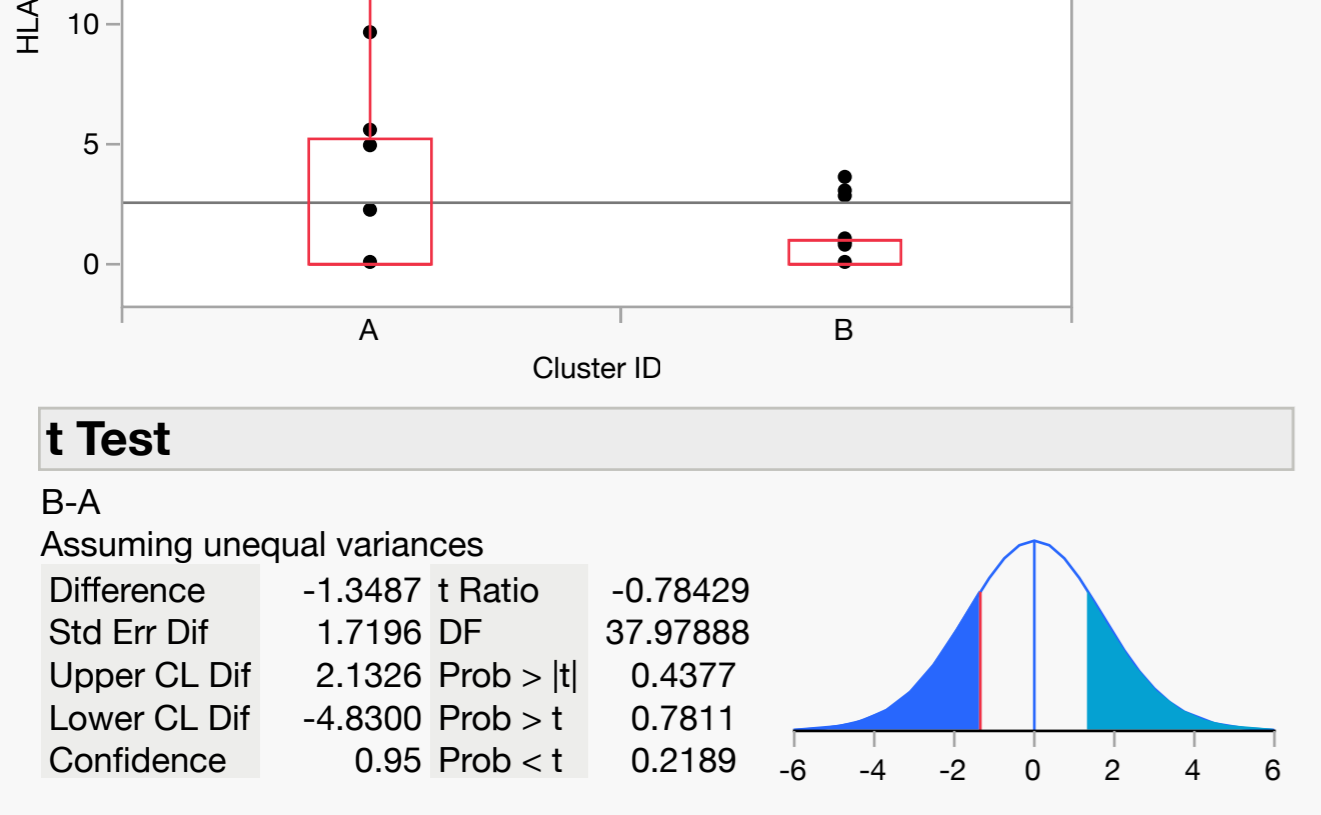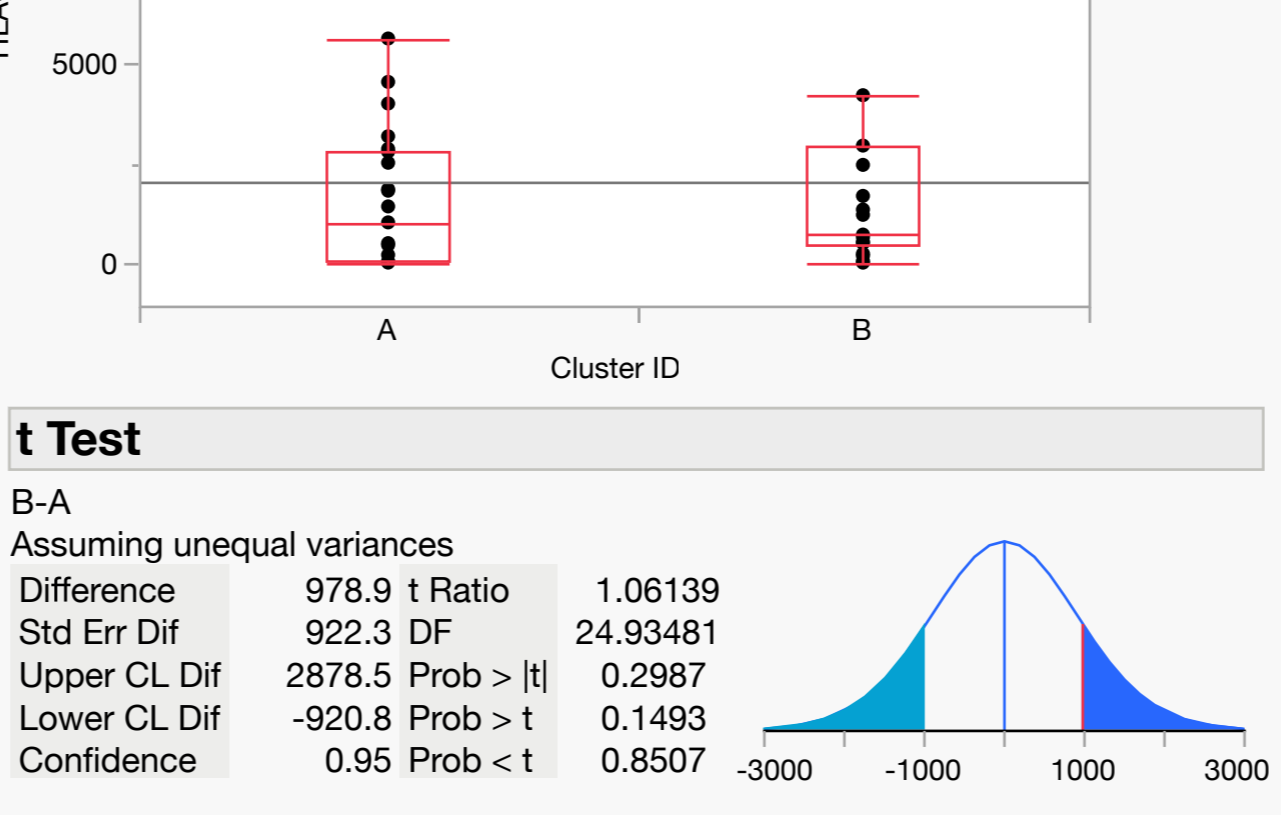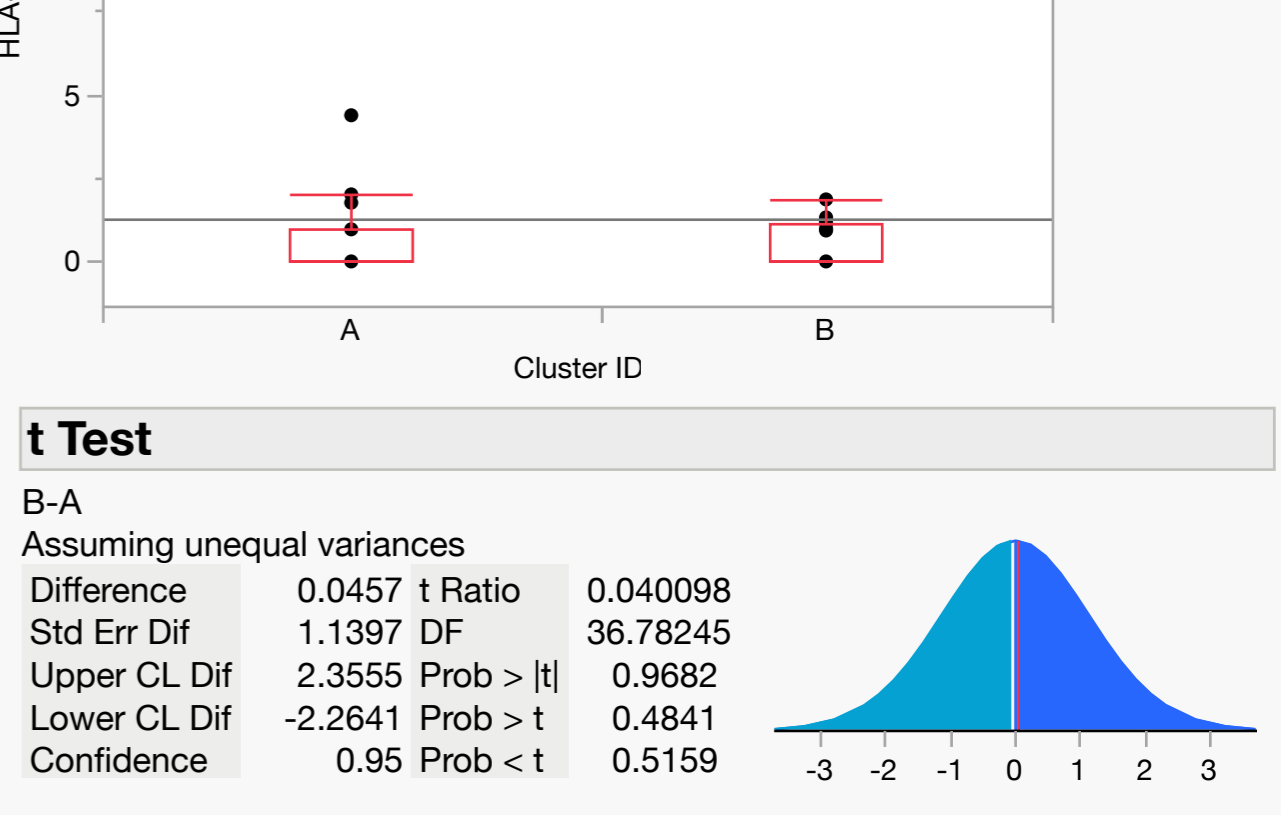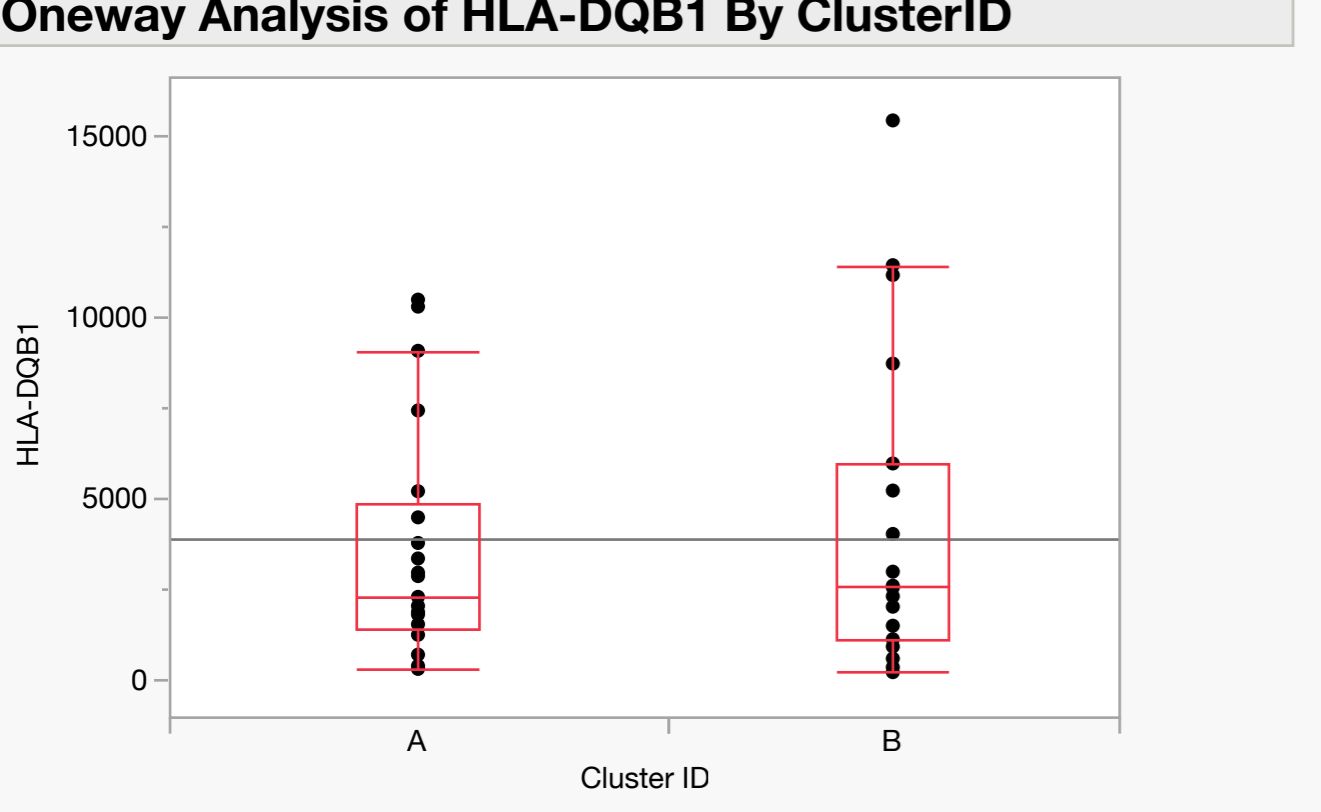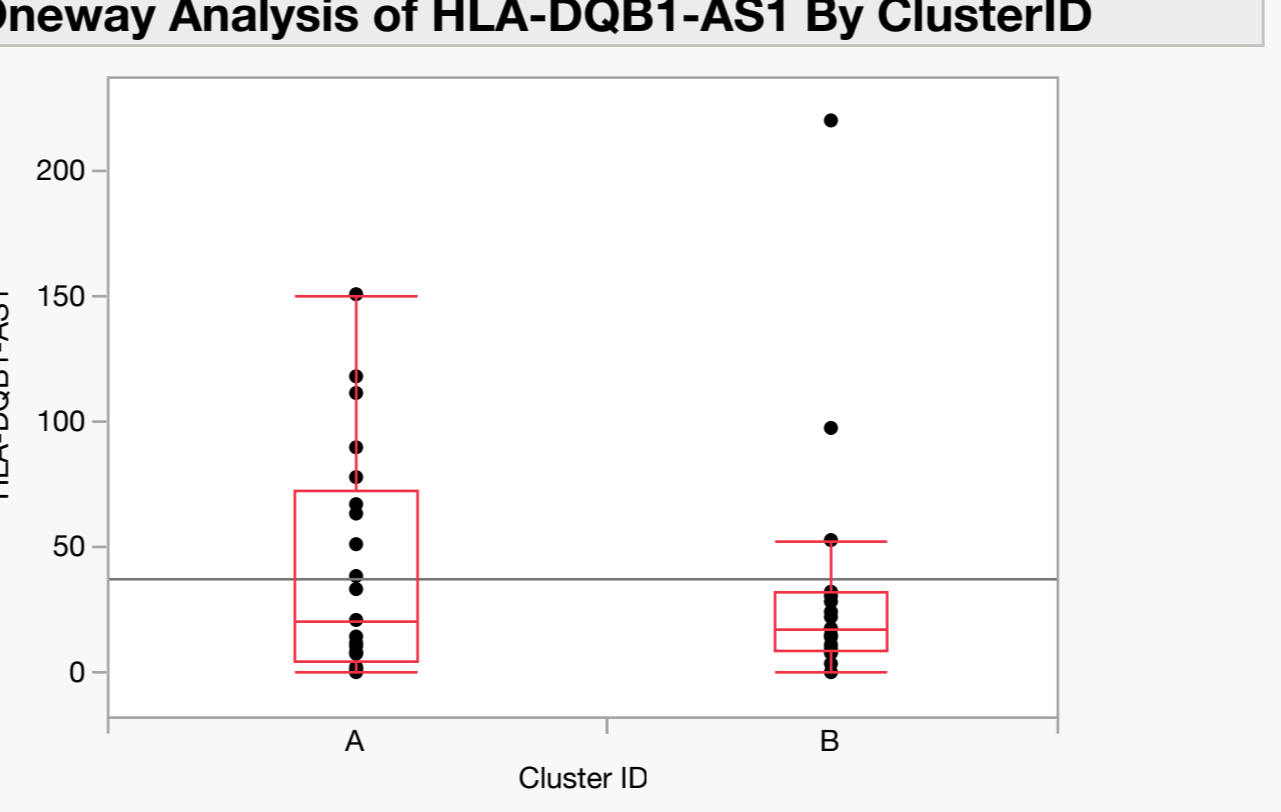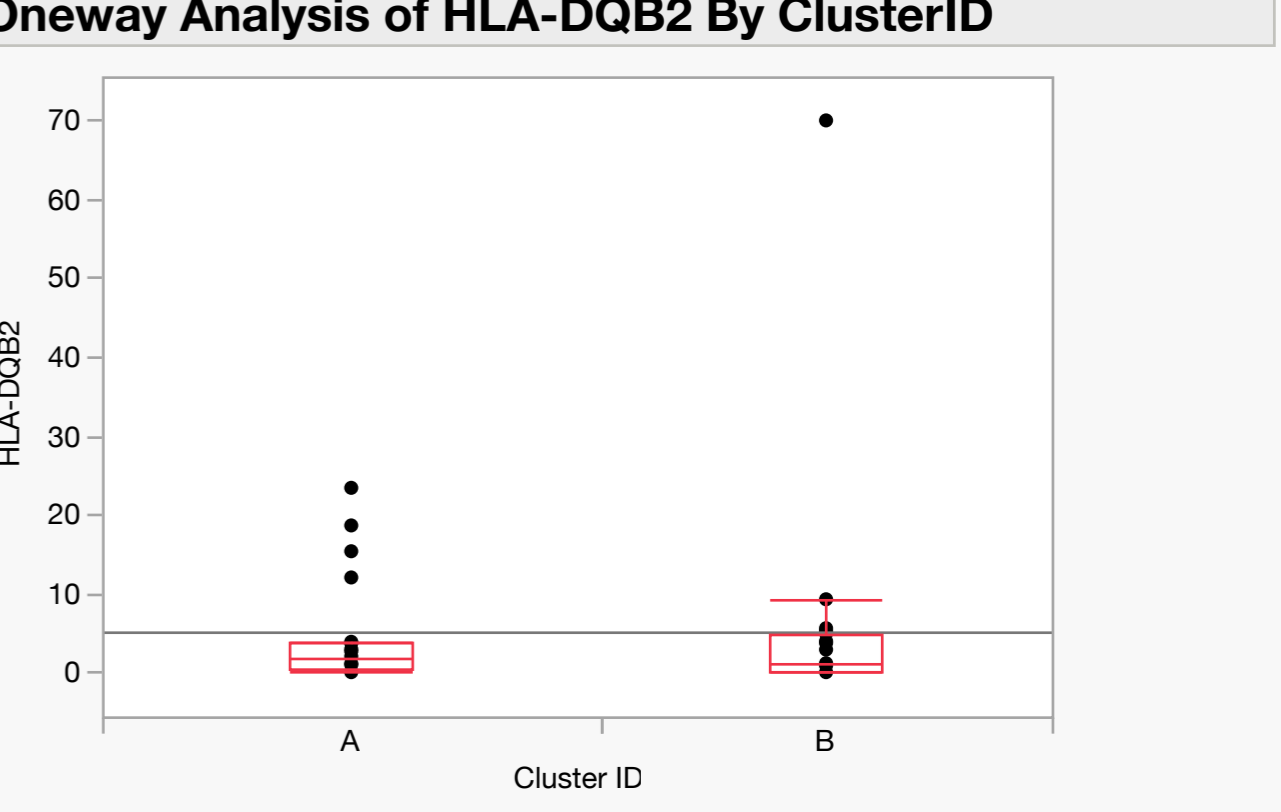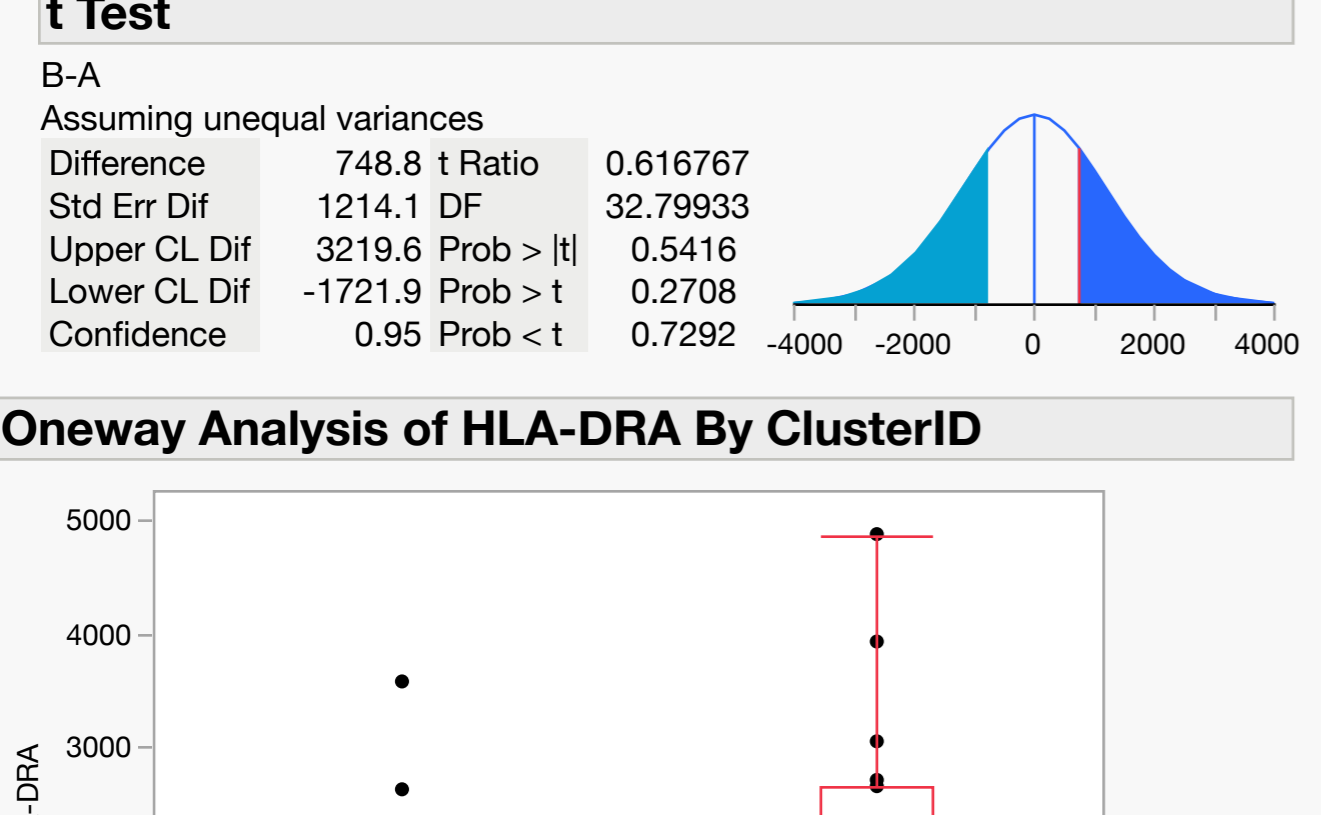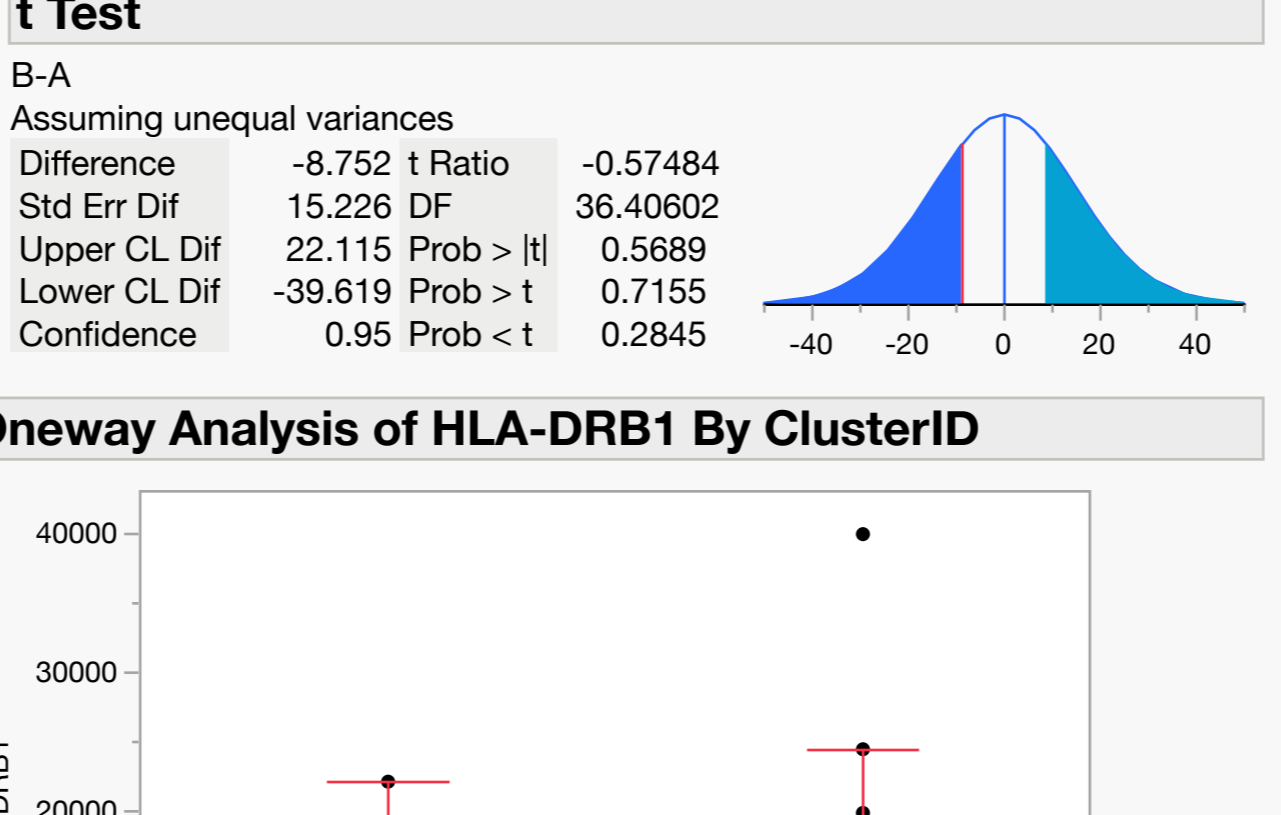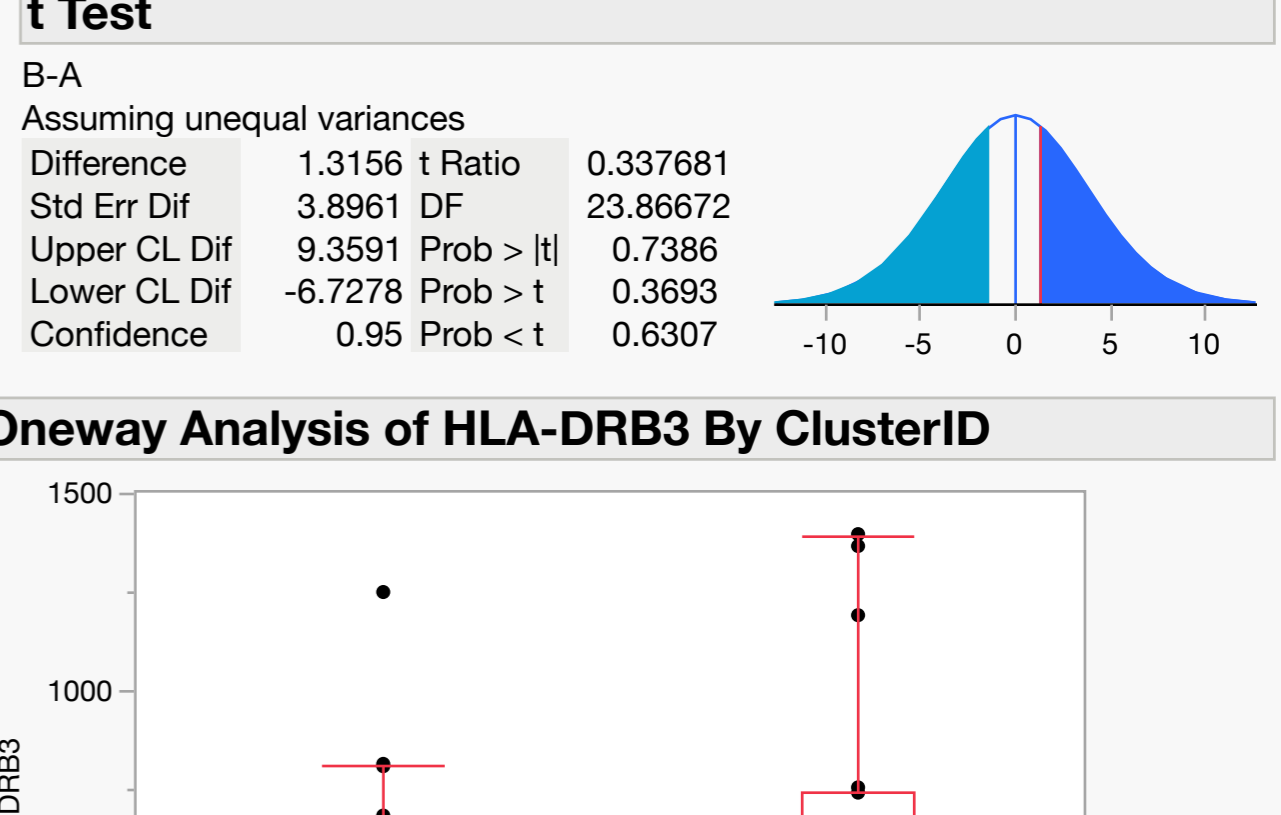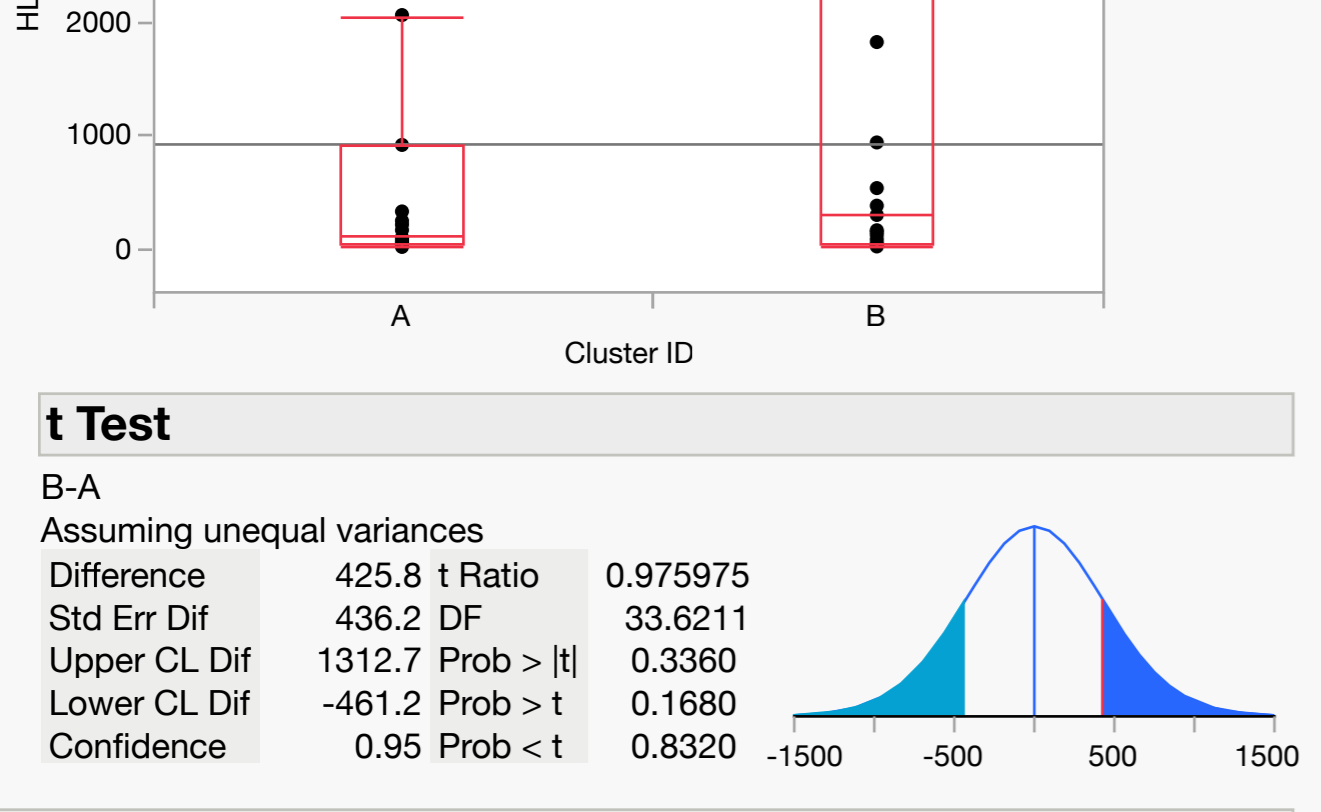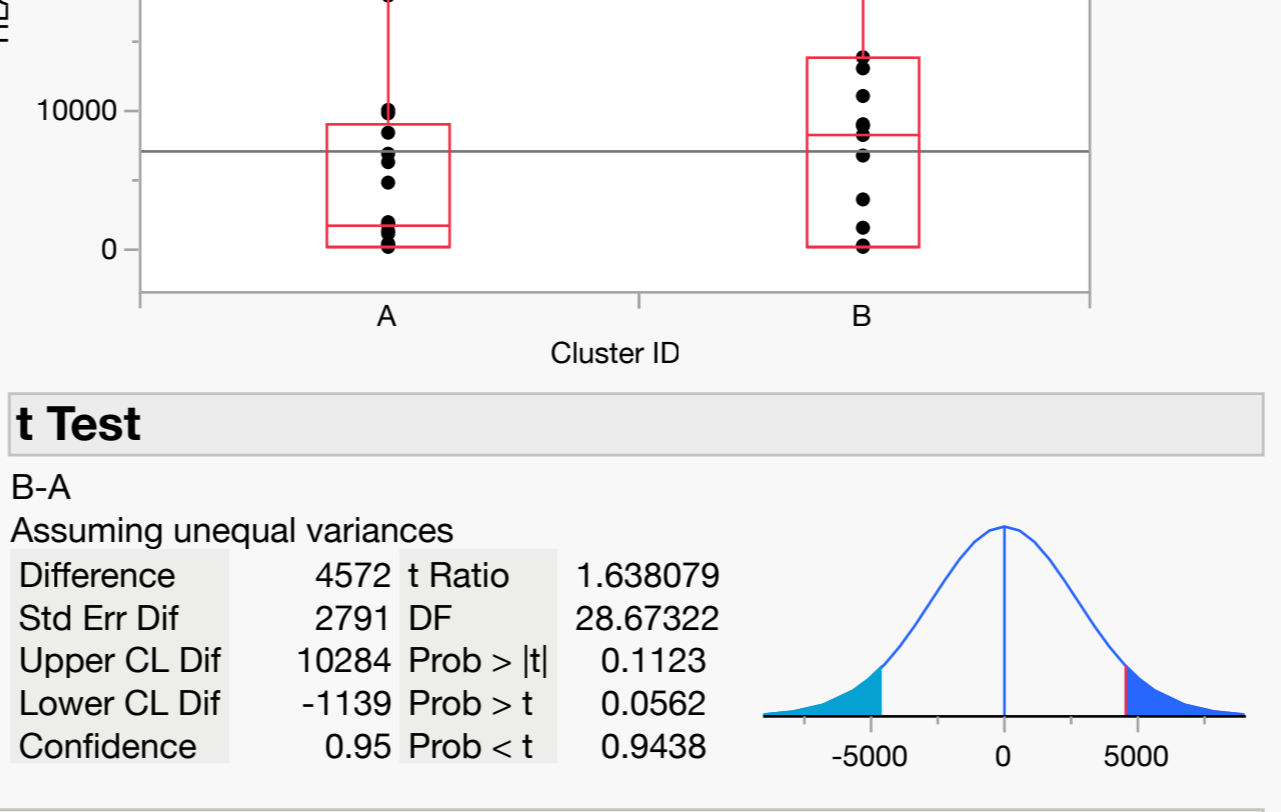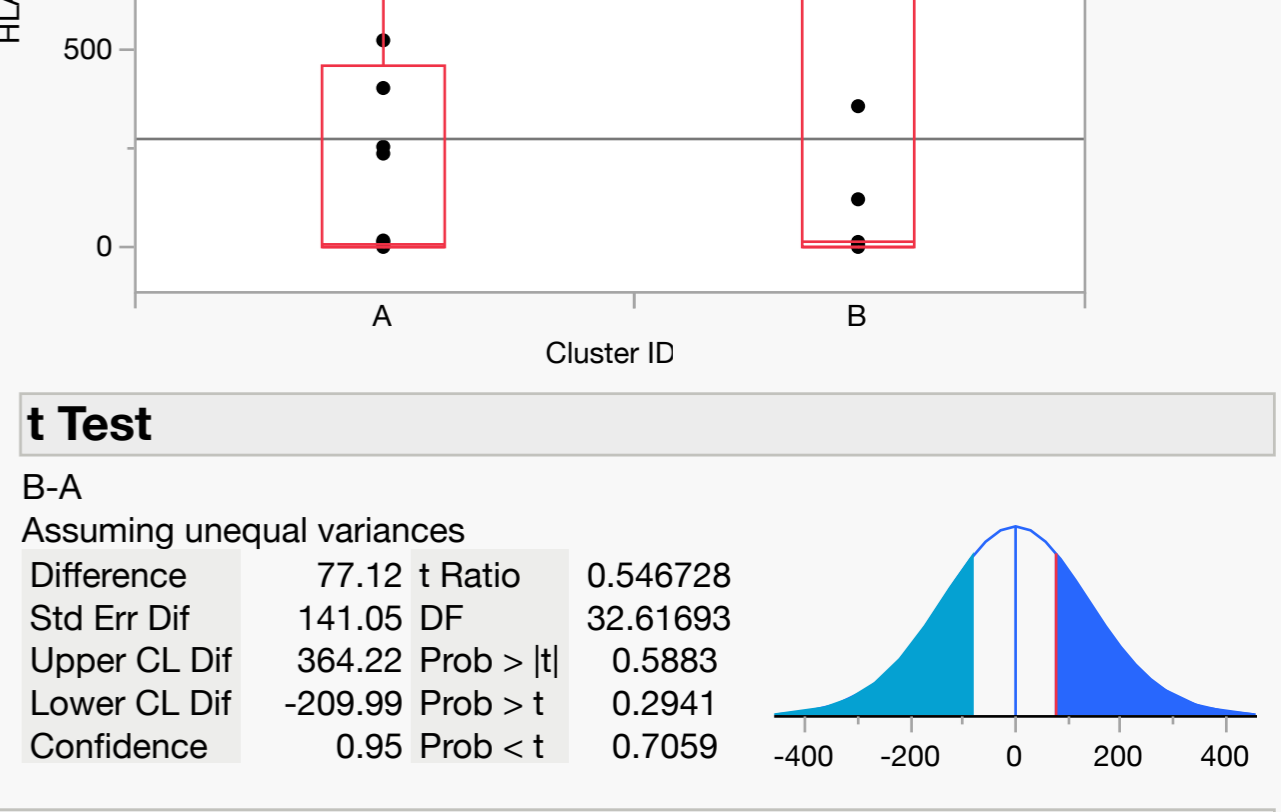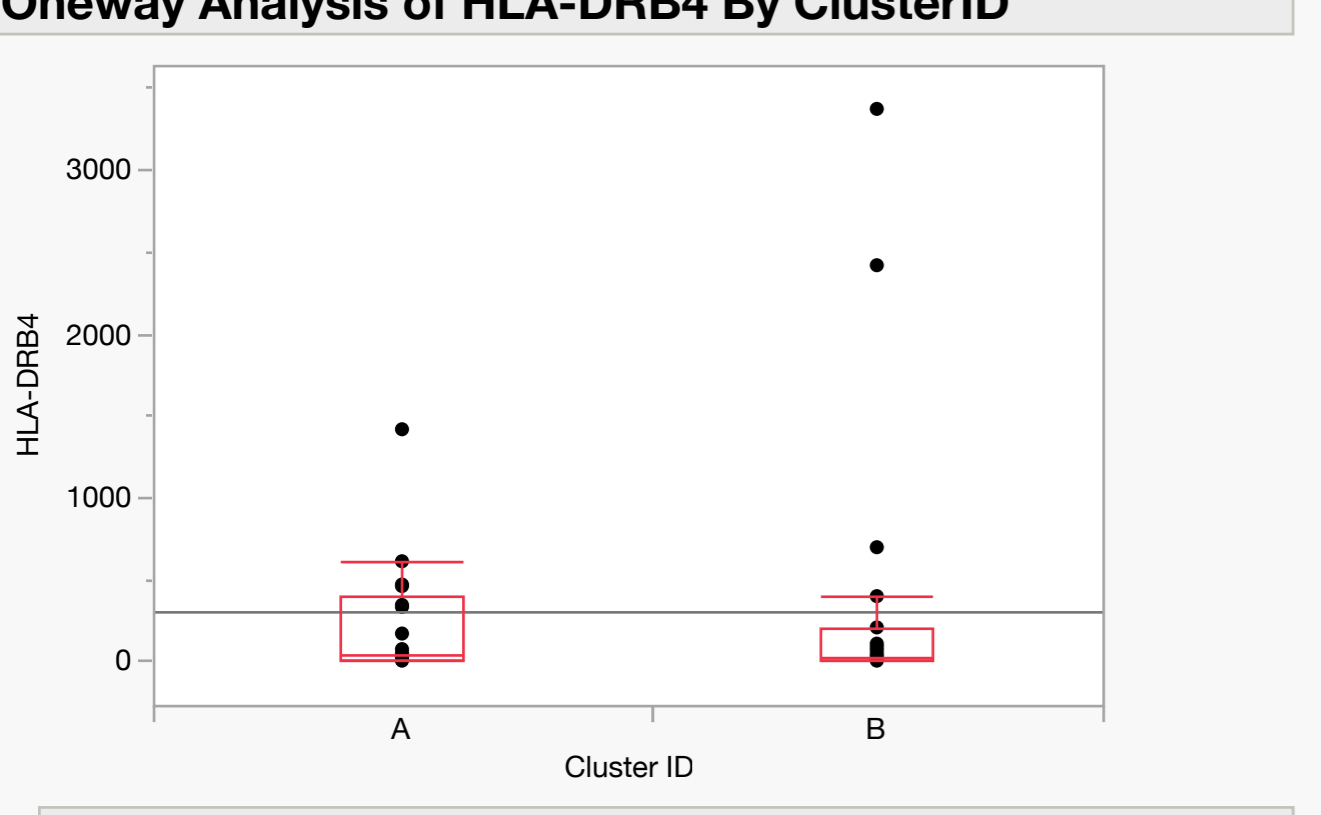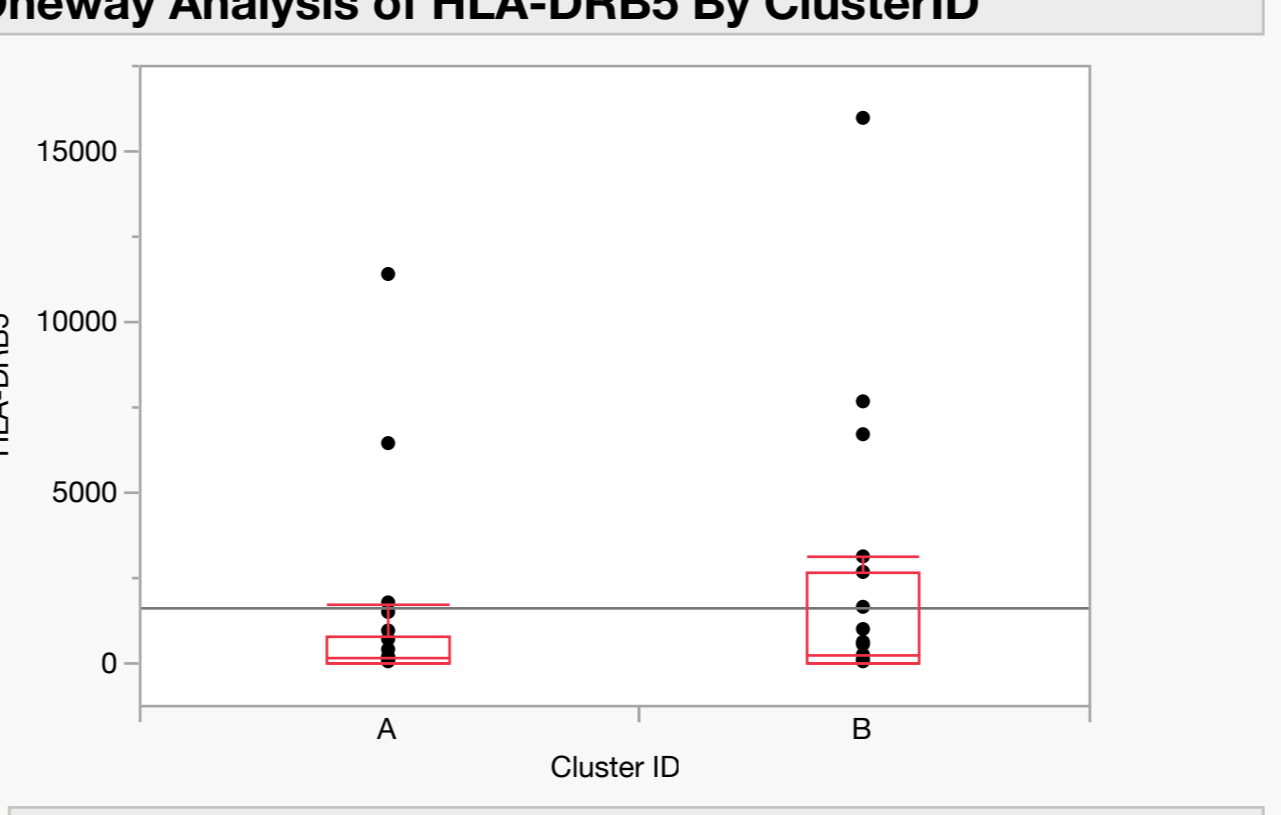

Supplement: Supplementary file 3 — Supplementary Material 3. [file 13046_2026_3790_MOESM3_ESM.pdf]

Asiedu et al.

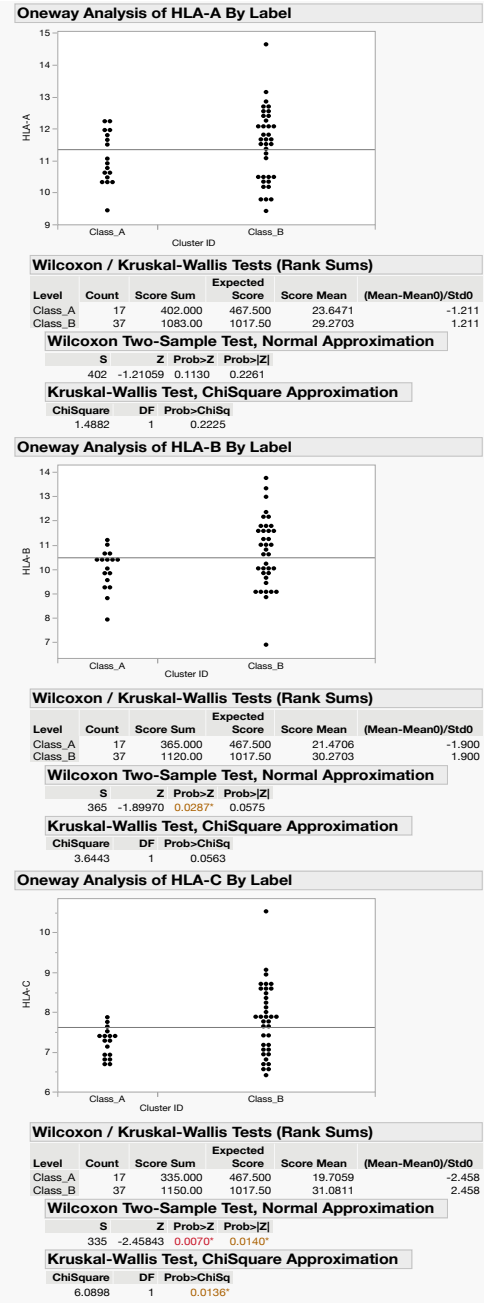

Laddha et al.

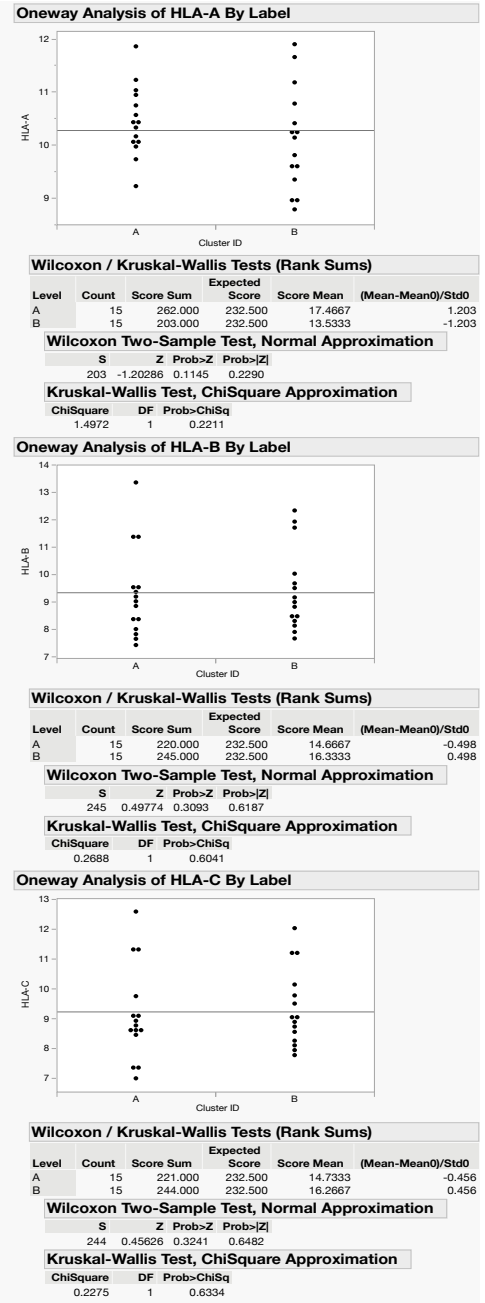

Supplement: Supplementary file 4 — Supplementary Material 4. [file 13046_2026_3790_MOESM4_ESM.pdf]
