# Supplementary material for: Transcriptional evidence of neuroendocrine cell plasticity beyond histological boundaries in lung neuroendocrine neoplasms: an in-silico analysis suggesting a progression model
Source: J Exp Clin Cancer Res. 2026 Jul 28;45:168. doi: 10.1186/s13046-026-03790-8 (PMC13418738; doi:10.1186/s13046-026-03790-8)
Supplement: Supplementary file 5 — Supplementary Material 5. [file 13046_2026_3790_MOESM5_ESM.docx]

| **Supplemental Table 1. Correlation analysis with LCNECs molecular subtypes** | | | | |
| --- | --- | --- | --- | --- |
|  |  |  |  |  |
| **George et al. SCLC (N=52)** | **P<0.0001*** |  |  |  |
| Subtype | **PNEN-A** | **PNEN-B** |  |  |
| SCLC-A | 36 (95%) | 2 (5%) |  |  |
| SCLC-N | 5 (100%) | 0 (0%) |  |  |
| SCLC-P | 0 (0%) | 7 (100%) |  |  |
| SCLC-Y | 0 (0%) | 2 (100%) |  |  |
| Na | 23 (79%) | 6 (21%) |  |  |
| *Likelihood Ratio Test |  |  |  |  |
|  |  |  |  |  |
| **George et al. LCNEC (N=40)** | **P=0.0166*** |  |  |  |
| Subtype | **PNEN-A** | **PNEN-B** |  |  |
| SCLC/SCLC-like | 6 (86%) | 1 (14%) |  |  |
| type 1 LCNEC | 10 (55%) | 8 (45%) |  |  |
| type 2 LCNEC | 2 (17%) | 10 (83%) |  |  |
| Na | 1 (33%) | 2 (67%) |  |  |
| *Likelihood Ratio Test |  |  |  |  |
|  |  |  |  |  |
| **Laddha et al. (N=30)** | **P<0.0001*** |  |  |  |
| Subtype | **PNEN-A** | **PNEN-B** |  |  |
| LC1 | 0 (0%) | 15 (100%) |  |  |
| LC2 | 8 (100%) | 0 (0%) |  |  |
| LC3 | 7 (100%) | 0 (0%) |  |  |
| *Likelihood Ratio Test |  |  |  |  |
